# Genome-wide identification and characterization of flowering genes in *Citrus sinensis* (L.) Osbeck: a comparison among *C. Medica* L., *C. Reticulata* Blanco, *C. Grandis* (L.) Osbeck and *C. Clementina*

**DOI:** 10.1186/s12863-024-01201-5

**Published:** 2024-02-20

**Authors:** Harleen Kaur, Pooja Manchanda, Gurupkar S. Sidhu, Parveen Chhuneja

**Affiliations:** School of Agricultural Biotechnology, College of Agriculture, Punjab Agricultural University, Ludhiana, 141001 Punjab India

**Keywords:** *Citrus*, Expression analysis, Flowering, Phylogeny, Synteny analysis

## Abstract

**Background:**

Flowering plays an important role in completing the reproductive cycle of plants and obtaining next generation of plants. In case of citrus, it may take more than a year to achieve progeny. Therefore, in order to fasten the breeding processes, the juvenility period needs to be reduced. The juvenility in plants is regulated by set of various flowering genes. The citrus fruit and leaves possess various medicinal properties and are subjected to intensive breeding programs to produce hybrids with improved quality traits. In order to break juvenility in *Citrus*, it is important to study the role of flowering genes. The present study involved identification of genes regulating flowering in *Citrus sinensis* L. Osbeck via homology based approach. The structural and functional characterization of these genes would help in targeting genome editing techniques to induce mutations in these genes for producing desirable results.

**Results:**

A total of 43 genes were identified which were located on all the 9 chromosomes of citrus. The in-silico analysis was performed to determine the genetic structure, conserved motifs, *cis*-regulatory elements (CREs) and phylogenetic relationship of the genes. A total of 10 CREs responsible for flowering were detected in 33 genes and 8 conserved motifs were identified in all the genes. The protein structure, protein-protein interaction network and Kyoto Encyclopedia of Genes and Genomes (KEGG) analysis was performed to study the functioning of these genes which revealed the involvement of flowering proteins in circadian rhythm pathways. The gene ontology (GO) and gene function analysis was performed to functionally annotate the genes. The structure of the genes and proteins were also compared among other *Citrus* species to study the evolutionary relationship among them. The expression study revealed the expression of flowering genes in floral buds and ovaries. The qRT-PCR analysis revealed that the flowering genes were highly expressed in bud stage, fully grown flower and early stage of fruit development.

**Conclusions:**

The findings suggested that the flowering genes were highly conserved in citrus species. The qRT-PCR analysis revealed the tissue specific expression of flowering genes (*CsFT*, *CsCO, CsSOC, CsAP, CsSEP* and *CsLFY*) which would help in easy detection and targeting of genes through various forward and reverse genetic approaches.

**Supplementary Information:**

The online version contains supplementary material available at 10.1186/s12863-024-01201-5.

## Introduction

Citrus plants undergo transition from vegetative meristem into floral meristem to induce flowering which is a fundamental life process required for the generation of progeny [1]. Flowering is regulated by various environmental and endogenous factors such as photoperiod, vernalization, high ambient temperatures, plant age, gibberellin concentration and plant’s carbohydrate profile [2, 3]. Flowering is induced when these factors are perceived in the leaves and shoot apical meristem by photoreceptors. The analysis of genetic and physiological parameters in *Arabidopsis thaliana* revealed that the flowering in response to the above mentioned factors is regulated by more than eighty genes [4].

The regulation of flowering occurs *via* complex network of four genetically regulated pathways [5]. Two of these pathways which mediate environmental responses are known as the long-day and vernalization pathways. The other two pathways functioning independent of environmental factors are the autonomous pathway and the gibberellin pathway [5]. The autonomous pathway promotes flowering under all conditions; whereas the gibberellin pathway functions under non-inductive short-day conditions. Some of these genes include *FLAVIN-BINDING*, *KELCH REPEAT, F-BOX1* (*FKF1*), *GIGANTEA* (*GI*), *CRYPTOCHROME2* (*CRY2*), *FLOWERING LOCUS* E (*FE)*, *CONSTANS* (*CO)*, and *FLOWERING LOCUS T* (*FT*) [6, 7]. Some of these genes are specific to regulate flowering while others are involved in perception of light signals. The genes *CRY, GI, FT*, and *CO* are majorly involved in photoperiod pathways [8, 9]. A superfamily of genes which encode Phosphatidylethanolamine-binding proteins (PEBP) is highly conserved across various taxa of prokaryotes, insects, mammals and plants [10, 11]. In case of plants, *PEBP* genes play fundamental role in regulating the time of flowering [12–14]. In angiosperms, PEBP family genes are grouped into three clades: *FT*, *TERMINAL FLOWER 1* (*TFL1*) and *MOTHER OF FT AND TFL1* (*MFT*) [15, 16]. The *MFT*-like genes have been reported to exist in both basal land and seed plants, while *FT*-like and *TFL1*-like genes have only been found in gymnosperms and angiosperms.

The mechanism of flowering has been well studied in case of *Arabidopsis* in which the flowering genes function in a sequential manner. The protein FT acts as a floral signal transducer which moves from leaves to the shoot apical meristem and promotes flowering [17]. In shoot apical meristem it interacts with *FLOWERING LOCUS D (FD)* to activate the downstream components of the flowering pathway [18]. On the contrary, the protein TFL1 helps in maintaining inflorescence meristem identity in shoot apex to inhibit flowering by competing with FT to bind with FD [19]. The balance between FT and TFL1 is necessary to modulate the floral transition and inflorescence architecture by affecting determinacy of meristem identity [12]. Besides these two proteins, the PEBP family genes *MOTHER OF FT AND TFL1 (MFT), TWIN SISTER OF FT (TSF), BROTHER OF FT AND TFL1 (BFT*), and *CENTRORADIALIS (CEN*) also function in regulating flowering [20]. The *MFT* gene functions in integrating the abscisic acid and gibberellic acid signalling pathways and acts in a PIF1-dependent manner repressing the seed germination under conditions of far-red light [14]. It weakly regulates flowering in *Arabidopsis* [21]. The *TSF* encodes a homolog of *FT* which induces flowering under conditions of non-inductive short days [22]. In *Arabidopsis*, the overexpression of repressors *BFT* and *CEN* resulted in a late flowering phenotype which was similar to plants overexpressing *TFL1* [16]. Similar functions of PEBP genes had also been reported in rice [23], tomato [24], apricot [25] and orchid [26]. The flowering genes are also known to exhibit tissue specific expression. The transcriptome analysis of *Arabidopsis* revealed that the genes regulating flower development were majorly expressed in reproductive parts of the plant and were characteristic to floral reproductive structures [27]. Thus, the identification of specific tissues showing high expression of genes is mandatory for directing to genetic engineering technologies.

Citrus fruits comprise the most important and extensively grown tree fruit crops globally. The genus consists of various species of pummelo, mandarin, citron and their hybrids such as sweet orange, grapefruit, lemon and lime. Citrus fruits are of high commercial value and are rich in antioxidants, micro- and macro-nutrients [28–32] which possess anti-inflammatory properties. The production of citrus orchards from seeds tends to take more than five years. Thus, the aim of the cultivators is to breakdown the long juvenile period, which poses challenges in genetic improvement of citrus [33, 34]. Different strategies are being adopted by researchers in order to reduce juvenile period, some of which include use of rootstocks, application of phyto-regulators and plant submission to the abiotic stresses [35]. Conventional methods of breeding such as crossing and clonal selection are long term processes. New approaches of biotechnology include virus induced flowering [36, 37], RNAi silencing [38–40], and CRISPR/Cas9 mediated knockout of flowering genes [41–46] which are associated with deep study of flowering genes. Hence, understanding is required of the mechanisms regulating flowering at genetic and molecular level for generating new prospects to reduce the vegetative period and consequently promote flowering.

The present study identified the genes which regulate flowering in *C. sinensis* L. Osbeck. The in-silico analysis of the genes was carried out to determine their genetic organization, conserved motifs, CREs and phylogenetic analysis, physical and chemical analysis of proteins. A heat map was generated to study the expression study of flowering genes in various tissues of different citrus species *viz., C. sinensis* (L.) Osbeck (sweet orange), *C. clementina* (clementine), *C. reticulata* Blanco (mandarin), *C. medica* L. (citron) and *C. grandis* (L.) Osbeck (pummelo). The *FT* genes identified in the present study could be used for inducing early flowering through transgenic approaches. It would provide information on genes which would help in paving new pathways for inducing early flowering in citrus, hence, accelerating citrus breeding programmes.

## Materials and methods

### Identification, sequence retrieval and intron-exon gene structure of flowering genes in sweet orange

The literature was reviewed to identify the genes which control flowering in different crops. Genomic, coding, cDNA and amino acid sequences of the flowering genes were retrieved from sweet orange genome through BLASTn using various databases (https://plants.ensembl.org/index.html, https://www.citrusgenomedb.org/, and https://www.ncbi.nlm.nih.gov/). Top hits with more than 80% identification and e-value ≤ e^−10^ were selected. The distribution of genes onto the nine chromosomes of sweet orange was performed using Phenogram Plot (http://visualization.ritchielab.org/phenograms/plot).

The organization of exonic and intronic regions of the flowering genes were identified using full length genomic and coding sequences of flowering genes using Gene-Structure Display Server GSDS2.0 (https://gsds.cbi.pku.edu.cn) [47].

### CRE analysis and identification of conserved motifs

The promoters of these genes were examined for the presence of CREs of flowering genes. The anti-sense and sense strands of region upstream of the transcription start site (ranging from 72 to 2117 bp) were analysed using Plant CARE (http://bioinformatics.psb.ugent.be/webtools/plantcare/html/) [48] and PLACE (https://www.dna.affrc.go.jp/PLACE/?action=newplace) [49]. The MEME suite (https://meme-suite.org/meme/tools/meme) was used to detect the conserved motifs [50] with the maximum number of motifs set at 8 with following parameters: motif width ranging from 6 to 50 and number of sites in sequences for each motif ranging from 2 to 200.

### Phylogenetic analysis

The amino acid sequences of *MADS* flowering genes from sweet orange, clementine, mandarin, citron, pummelo, *Arabidopsis thaliana, Brassica rapa* (brassica), *Musa acuminata* (banana), *Citrullus lanatus* (watermelon) and *Ananas comosus* (pineapple) were aligned using ClustalW. The phylogenetic relationship was determined with a model organism (*Arabidopsis*), and monocots (banana and pineapple) and dicots (brassica and watermelon) using the Maximum-Likelihood method with bootstrap test of 1000 replicates using MEGA XI software [51]. The phylogenetic tree was conceptualized through iTOL Interactive Tree of Life (https://itol.embl.de/).

### Gene ontology (GO) analysis and KEGG pathway annotation

The analysis of functional and annotation data of flowering genes was performed via BLAST2GO tool (https://www.blast2go/com/) [52]. The proteins sequences were subjected to BLASTP against protein database of NCBI (https://blast.ncbi.nlm.nih.gov/Blast.cgi) followed by mapping and retrieval of GO terms and then annotation of GO terms. The results were categorized as ‘Cellular components’, ‘Biological processes’ and ‘Molecular functions’ according to which the GO terms were assigned. In addition to this, the KEGG mapping (https://www.genome.jp/kegg/) [53] was performed to elucidate the functions in which the flowering genes participate as enzymes. To generate more comprehensive data, the ‘Gene Function’ of the flowering related genes was retrieved from Citrus Pan-genome to Breeding database (http://citrus.hzau.edu.cn/geneFunc/query.php). The analysis was performed in ‘Gene Function’ module using following sources: CDD, Gene3D, Hamap, PANTHER, Pfam, PIRSF, PRINTS, ProSitePatterns, ProSiteProfiles, SFLD, SMART, SUPERFAMILY, and tigrfam.

### Physical and chemical properties, homology modelling and protein-protein interaction (PPI) network

The physical and chemical properties of the proteins encoded by flowering genes were determined via ProtParamExPasy server (https://web.expasy.org/protparam/) [54]. The properties included amino acid length, molecular weight, instability index, PI value, aliphatic index and Grand Average of Hydropathicity index (GRAVY). ProtComp version 9.0 server (http://www.softberry.com/) was used to determine the sub-cellular localization of the proteins and the Pfam domains were predicted via Pfam 35.0 (http://pfam.xfam.org/) based on profile of Hidden Markov Models [55]. For predicting the protein structure, the amino acid sequences were submitted to Phyre2 (Protein Homology/analogY Recognition Engine; http://www.sbg.bio.ic.ac.uk/phyre2) under ‘expert’ mode [56]. The amino acid sequences were submitted to STRING v11.5 (https://string-db.org/) with confidence level medium (0.400) and false discover rate stringency of 5% for the generation of PPI model.

### Comparative genomics and synteny analysis

The genome databases of five species of citrus viz., sweet orange, clementine, mandarin, citron, and pummelo were compared. The data was retrieved from Citrus Genome Database (https://www.citrusgenomedb.org/). The differences in structures of major genes and proteins were analysed *via* GSDS2.0 (https://gsds.cbi.pku.edu.cn) and Phyre2 (http://www.sbg.bio.ic.ac.uk/phyre2). Citrus genome database was explored to perform the synteny analysis to observe collinearity between *C. sinensis, C. maxima*, and *C. clementina* genomes.

### Expression analysis of flowering genes

The expression analysis of flowering genes was evaluated in different tissues (ovule, fruit, fruit peel and floral bud) of various citrus species (*C. reticulata, C. unshiu*, and *C. clementina*). The rkpm values were retrieved from Citrus Pan-genome to Breeding database (http://citrus.hzau.edu.cn/index.php) and the heat map was constructed using R package. To retrieve rkpm values, the individual gene ids for each species were fed to ‘Gene Expression Search’ using pipeline TopHat2 + Cufflinks.

### qRT-PCR analysis of flowering genes

The fold change in expression level of flowering genes was performed using qRT-PCR analysis. The various tissues of sweet orange were collected which included leaf, bud, flower and fruit stages (FruitS1: 7–8 days after flowering; FruitS2: 30–32 days after flowering; and FruitS3: 60–70 days after flowering) to study the gene expression of *CsFT, CsCO, CsSOC, CsAP, CsSEP*, and *CsLFY*. Furthermore, the leaf tissues from various species (*C. sinensis, C. unshiu, C. clementina* and *C. reticulata*) were used to compare the expression level of flowering genes within these species. The total RNA from the samples was extracted using Trizol™ reagent method and cDNA was synthesized using PrimeScript 1st strand cDNA synthesis kit (Takara Bio Inc.). The primers used in the study were designed using PerlPrimer software (v1.1.21). The lists of primers used in the study are given in Additional file 1: Table S1. The qRT-PCR analysis was performed using GoTag qPCR Master Mix (Promega Corp.) by taking *CsACTIN* as internal control. The fold change in relative gene expression was calculated using method given by Livak and Schmittgen [57]. The experiment was performed using three biological replicates and three technical replicates.

## Results

### Determination of chromosomal location and genetic organisation of flowering genes

A total of 43 genes were identified in *C. sinensis* genome. The sequences were retrieved *via* BLASTN. The genes included *FT, CO, SOC1, BFT, TFL, SVP* (*SHORT VEGETATIVE PHASE*), *MAF1* (*MADS AFFECTING FLOWERING*), *MADS* genes (*MADS_AGL31, MADS_AGL61, MADS_AGL70, MADS_AGL3, MADS_AGL35, MADS_AGL42, MADS_AGL82* and *MADS_AGL72*), *SHP1* (*SHATTERPROOF*), *GI, AP* (*APETALA*, *AP2* and *AP3*), *PHYB* (*PHYTOCHROME*), *CRY* (*CRYPTOCHROME*; *CRY1* and *CRY2*), *WUS* (*WUSCHEL*), *FLD* (*FLOWERING LOCUS D*), *FLK* (*FLOWERING LOCUS K*), *DL4* (*DROOPING LEAF*), *TSF* (*TWIN SISTER OF FT*), *PI* (*PISTILLATA*), *LFY* (*LEAFY*), *FLC* (*FLOWEING LOCUS C*), *FRI* (*FRIGIDA*), *EMF* (*EMBRYONIC FLOWER*), *CEN* (*CENTRORADIALIS*), *TEM1* (*TEMPRANILLO1*), *FT3* (*FLOWERING LOCUS T3*), *SPB* (*SQUAMOSA PROMOTER BINDING*), *SPL* (*SQUAMOSA PROMOTER BINDING LIKE*), *SUF* (*SUPPRESSOR OF FRI*), *VIN* (*VERNALIZATION INSENSITIVE*), *VIP* (*VERNALIZATION INDEPENDENCE*), *DELLA*, and *ZTL* (*ZEITLUPE*). All the genes regulate flowering (either via induction or inhibition) at different stages of plant growth and under different conditions. *FT*, *SOC1*, and *LFY* act as the primary genes which are responsible for integrated induction of flowering [58]. *AP1* and *LFY* along with E-class *SEP* genes are type of floral meristem identity genes which play a key role in regulation of flowering pattern [59]. The gene *LFY* acts in association with *AP* to promote the metamorphosis from inflorescence to floral meristem [60]. The *MAF* genes delays the flowering time with its overexpression [61]. The *WUS* gene promotes structural and functional integrity in indeterminate shoot and determinate floral meristems [62]. *FLC* encodes a MADS-BOX transcription factor which acts by repressing the expression of *FT* and *SOC1* in *Arabidopsis* [63]. An ortholog of *FLC, CcMADS19* represses the expression of *FT* in the leaf tissues [64, 65], whereas, *FRI* is a positive regulator of *FLC* [66]. During embryogenesis, *FRI* adjusts the expression level of *FLC* via chromatin modification which is helps ensuring flowering under vernalization conditions in new generation coming from vernalized parents [67]. A homolog of *CsTFL1*, *CsCEN* interacts with CsFLD in axillary meristems where it is expressed. The indeterminate co-expression of *CsCEN* and *CsFD* suggests their role in regulation of axillary bud development [68]. *EMF* genes regulate flowering time by maintenance of vegetative phase [69]. Disruption of *EMF* activity results in transgenic plants which exhibit flowering at different times. Studies have revealed that non-functional *EMF1* and/or *EMF2* genes results in flowering upon germination by omitting vegetative growth [70]. *VIN3* encodes a chromatin remodelling protein which functions under low temperatures [71]. This gene represses *MAF1* in response to vernalization [72] and *MADSAGL19* for the cold induction [73]. ZTL is a F-box circadian protein whose altered expression results in rate-dependent circadian period effects and causes changes in flowering time [74].

**Fig. 1:**
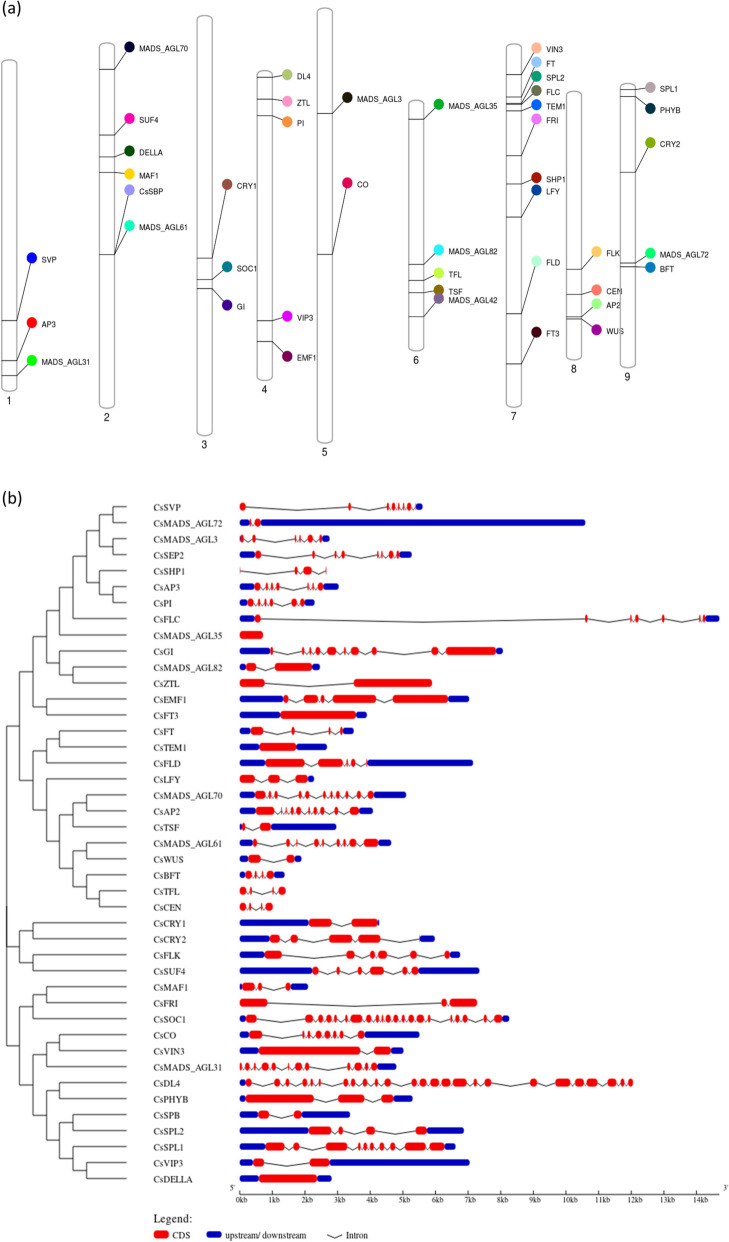
**a** Distribution of flowering genes on sweet orange (Cs) chromosomes (numbered 1–9). **b** Intron-exon structure of flowering genes (fit to scale). Red rectangles and thick black curved lines represent exons and introns, respectively

The genome-wide identification of the genes revealed the distribution of genes across all the chromosomes in sweet orange (Fig. 1a). The maximum number of genes (10) were located on chromosome 7 followed by chromosome 2 with six genes. The chromosomes 4, 6 and 9 had five genes; whereas chromosomes 1 and 3 had three genes each. Chromosomes 5 and 8 had two and four genes, respectively. The genes on chromosomes were clustered at either of the ends except chromosomes 2, 7 and 9 where the genes were distributed across the length of the chromosome. The organization of the introns and exons gave insights into the genetic structure of the genes. Of all, four genes were found to be intron-less which included *CsMADS_AGL35*, *CsTEM1*, *CsFT3* and *CsDELLA*. The genes *CsTSF*, *CsWUS*, *CsMADS_AGL82*, *CsCRY1*, *CsVIN3*, *CsSPB*, and *CsVIP3* had single intron which separated the coding sequence flanked by upstream and downstream sequences (Fig. 1b). Rest of the genes had coding sequences interrupted by numerous introns wherein *CsDL4* had the maximum number of introns followed by *CsSOC1*. The genes *CsFT* and *CsBFT* had similar arrangement of introns and coding sequences except for the sizes of the sequences which were less in the case of *CsBFT*.

### Identification of CREs and conserved motifs

**Fig. 2:**
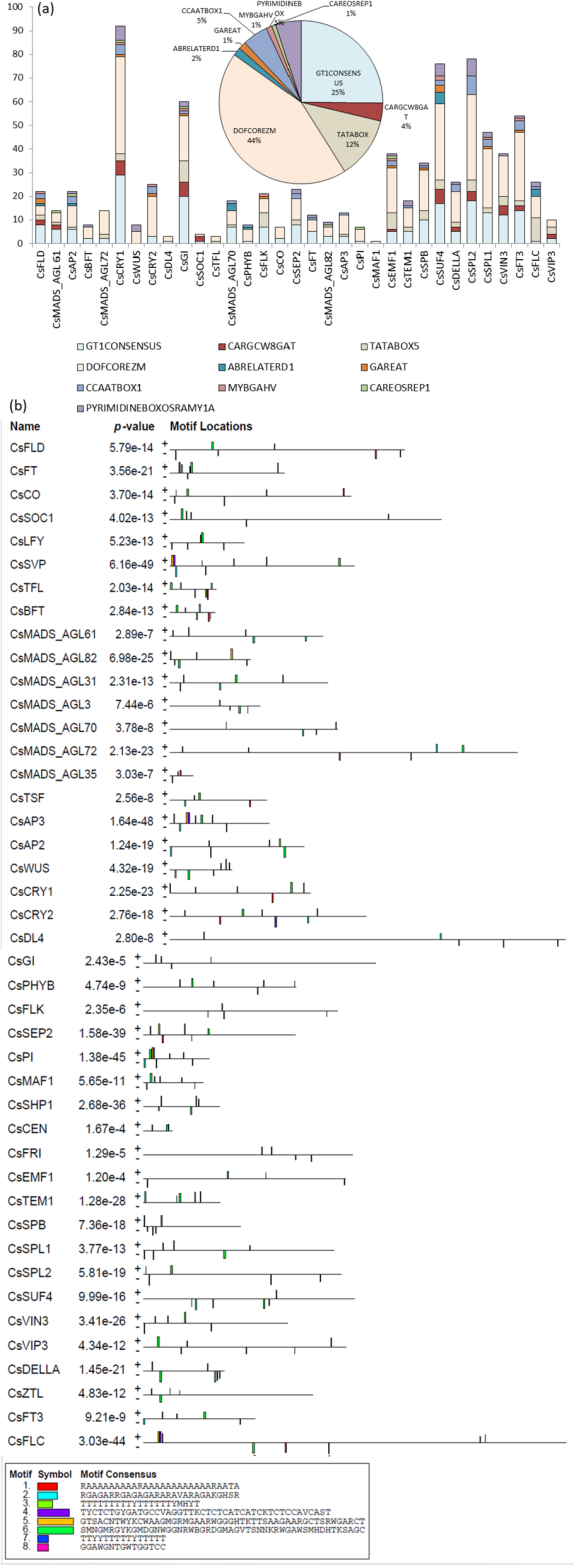
**a** Distribution of CREs on each flowering related gene (Insert: Abundance of CREs in flowering genes of sweet orange). **b** Various conserved motifs detected in nucleotide sequences of flowering genes in sweet orange shown in different colours

The regulation of gene expression is controlled either *via* transcription activation or repression. The molecular mechanism behind the regulation is the binding of transcription factors to their corresponding CREs which are located upstream of the genes (regions called promoters). These transcriptional factors can act as activator or repressor of the genes thereby, increasing or decreasing the expression of genes, respectively. Thus, the CREs play an important role in gene regulation. The CREs were identified in the promoter regions of the flowering genes. The different CREs and their location on 33 gene sequences are shown in Additional file 1: Table S2. A total of 10 different CREs were identified which included: GT1CONSENSUS (GRWAAW), CARG box (CWWWWWWWWG), TATA box, DOFCOREZM (AAAG), CCAAT box, ABRELATERD1 box (ACGTG), GARE box (TAACAAR), MYBGAHV (TAACAAA), Pyrimidine box (CCTTTT / TTTTTTCC) and CARE box (CAACTC). The TATA box was present as TATA box2 (TATAAAT), box4 (TATATAA), box5 (TTATTT), TATABOXOSPAL (TATTTAA) and TATAPVTRNALEU (TTTATATA). The DOFCOREZM and GT1CONSENSUS were the most common CREs present in the genes. The distribution and abundance of CREs is shown in Fig. 2a.

The conserved motifs were analysed in the flowering genes via MEME suite. A total of 8 motifs were identified, which were present on both positive and negative strand of the genes (Fig. 2b). The length of the motifs ranged from 15 to 50. The sequences of the motifs are shown in Fig. 2b. The genes *CsSVP*, *CsAP3*, *CsPI* and *CsFLC* had all the motifs. The motifs 4 and 5 were present as a single cluster in all these genes including *CsPI*. The gene *CsCEN* had motifs only on the positive strand. In rest of the genes, motifs were present on both the strands.

### Phylogenetic analysis

**Fig. 3:**
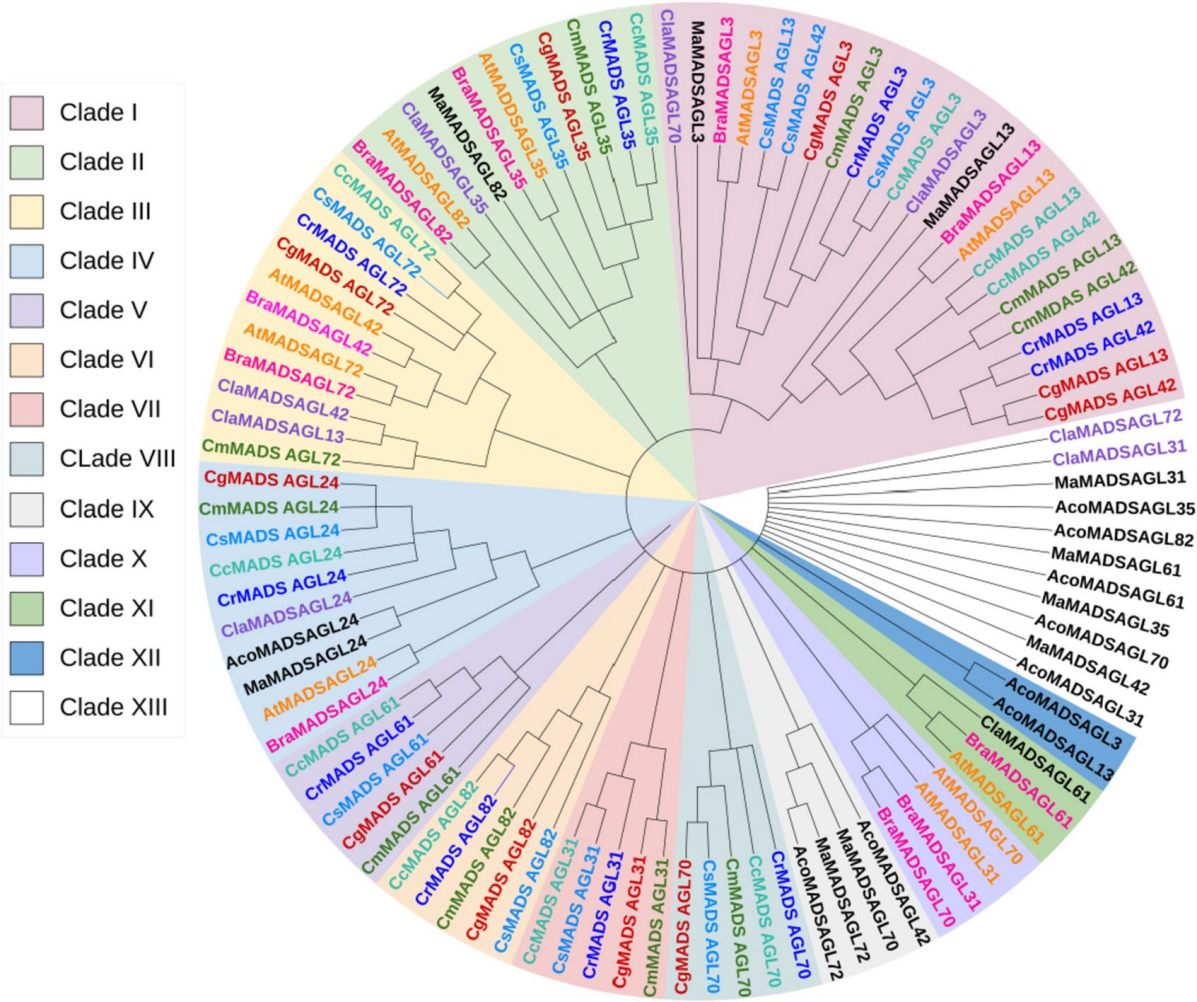
Phylogenetic tree showing relationship between flowering related MADS box AGL genes from Arabidopsis (At), citron (Cm), mandarin (Cr), sweet orange (Cs), pummelo (Cg), clementine (Cc), brassica (Br), watermelon (Cla), banana (Ma) and pineapple (Ac) denoted by different colours

The phylogenetic tree was constructed for MADS box *AGL* (*AGAMOUS LIKE*) genes in citrus species along with watermelon, brassica, banana, pineapple and *Arabidopsis*. The genes were divided into 12 clades and most of the genes of banana and pineapple were placed outside the clades (Fig. 3). The Clade I included *AGL13, AGL42* and *AGL3* genes of all species along with *AGL70* of watermelon. The *AGL3* of pineapple was present outside the clade. The Clade II had *AGL35* genes clustered with *AGL82* genes of banana, watermelon, Arabidopsis and brassica. The rest of the *AGL g*enes were present as separate Clade VI. Interestingly, *AGL24* gene of all the species were present in a single Clade IV depicting the conservation of gene during evolutionary process.

### GO annotation

**Fig. 4:**
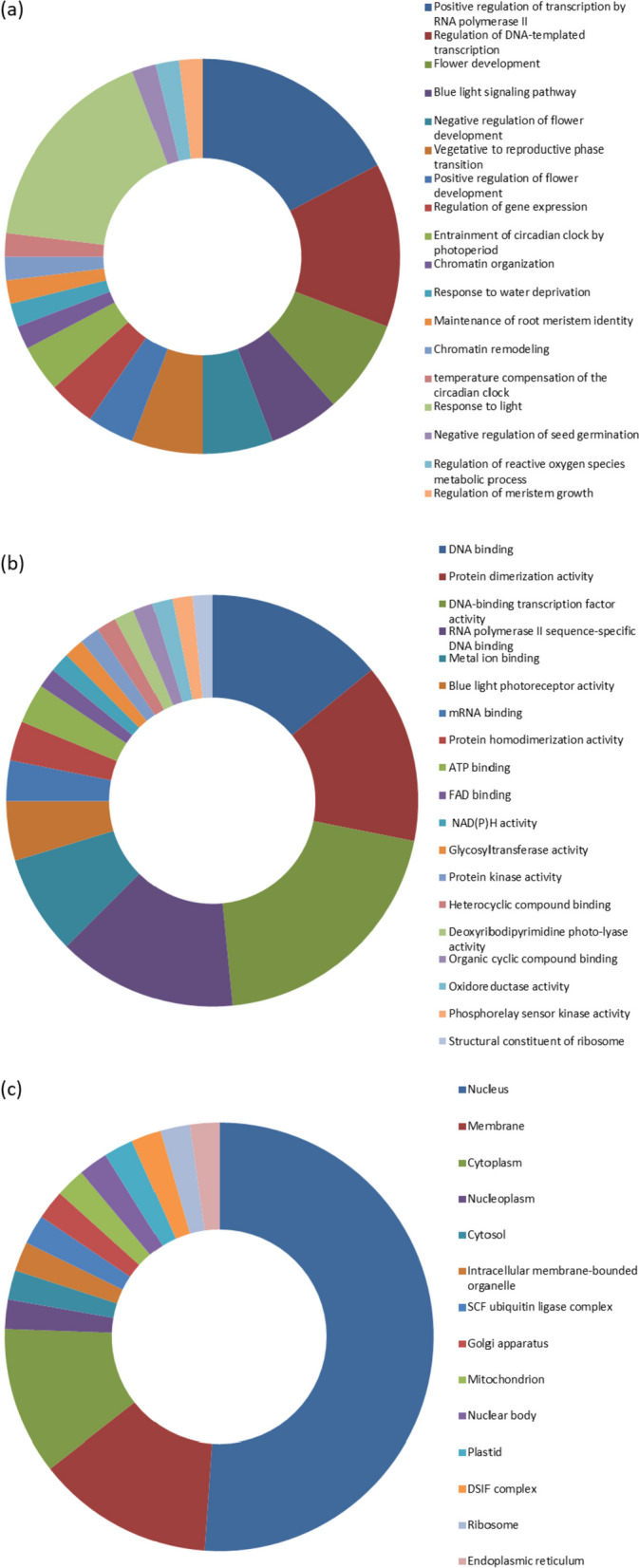
Distribution of genes into three categories **a** biological processes, **b** molecular functions and **c** cellular component via gene ontology analysis

The protein sequences of the flowering related genes were functionally annotated categorizing them into three categories based on ‘Cellular component’, ‘Molecular function’, and ‘Biological process’ (Fig. 4 and Additional file 1: Table S3). In case of ‘Biological process’, majority of the genes were involved in ‘Positive regulation of transcription’ (P:GO:0045944). In case of ‘Molecular function’, most of the sequences were annotated as involved in ‘Protein dimerization activity’ (F:GO:0046983). The sequences were annotated based on the cellular location. Most of the genes were located in nucleus (C:GO:0005634) followed by membrane (C:GO:0005886).

The GO annotation data was compared with the results retrieved from the ‘Gene function’ module of the Citrus pan-genome to breeding database (Additional file 1: Table S4). In case of ‘Biological process’, the genes were involved in ‘Cellular metabolic process’ (GO:0044237), ‘Metal ion transport’ (GO:0030001). In case of ‘Molecular function’, most of the sequences were annotated with ‘Protein dimerization activity’ (GO:0046983), ‘DNA binding’ (GO:0003677), ‘Protein binding’ (GO:0005515), ‘Monooxygenase activity’ (GO:0004497), ‘Oxidoreducatse activity’ (GO:0016705). Based on the ‘Cellular component’, the proteins were annotated under GO terms ‘Nucleus’ (GO:0005634) and ‘Membrane’ (GO:0016020). The results of the ‘Gene function’ analysis were in conformity with the GO annotation data (Fig. 4).

### Physical, chemical and structural properties of the proteins and their PPI network

**Fig. 5:**
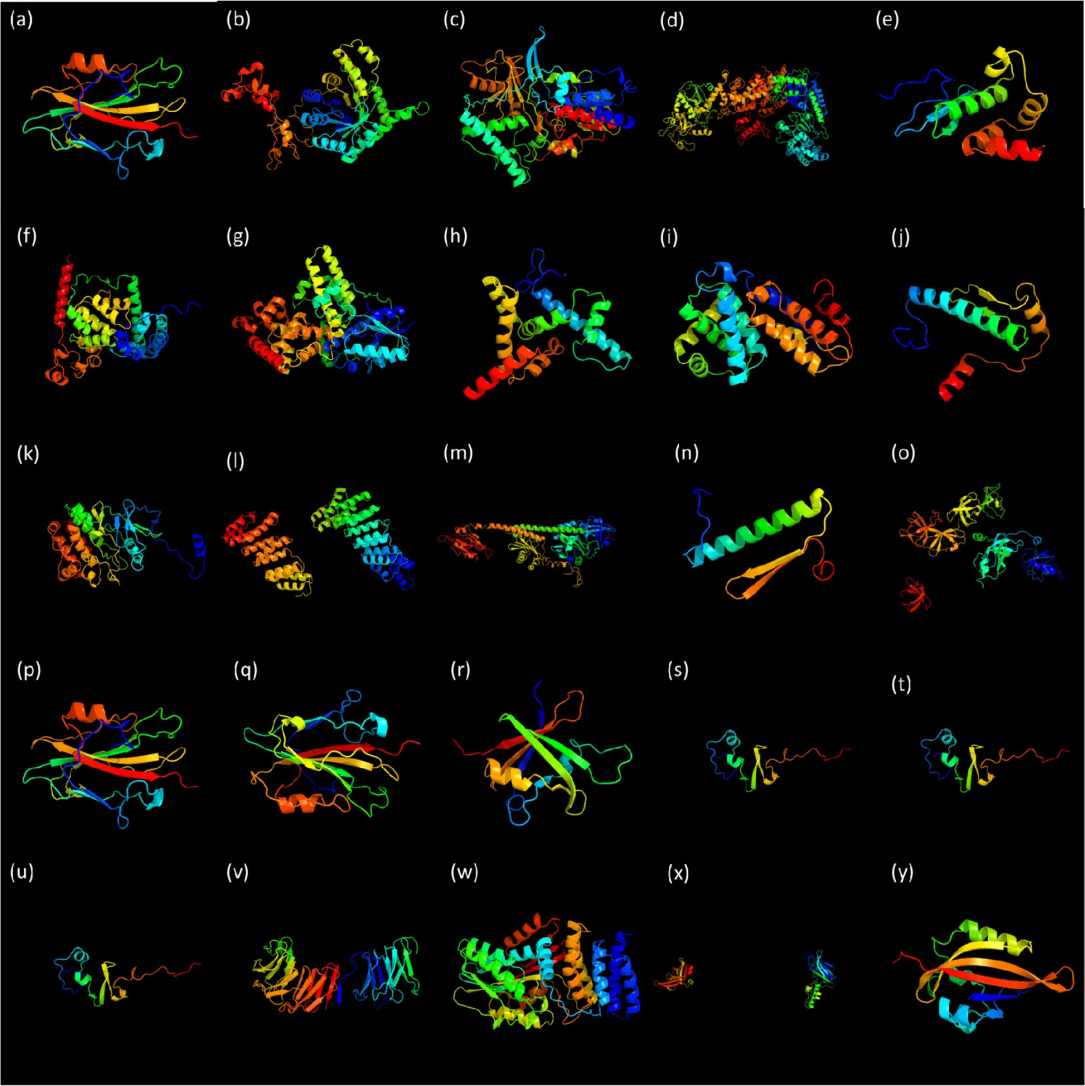
Protein structure of predicted with 100% confidence level (**a**) CsBFT (**b**) CsCO (**c**) CsFLD (**d**) CsDL4 (**e**) CsAP2 (**f**) CsCRY1 (**g**) CsCRY2 (**h**) CsLFY (**i**) CsMADS_AGL31 (**j**) CsMADS_AGL35 (**k**) CsMADS_AGL70 (**l**) CsMADS_AGL82 (**m**) CsPHYB (**n**) CsSHP1 (**o**) CsSOC1 (**p**) CsTFL (**q**) CsCEN (**r**) CsTEM1 (**s**) CsSPB (**t**) CsSPL1 (**u**) CsSPL2 (**v**) CsVIP3 (**w**) CsDELLA (**x**) CsFT3 (**y**) CsZTL

The physical and chemical properties of the proteins were determined using ProtParam expasy server. The lengths of the proteins ranged from less than 100 amino acids to more than 1500 amino acids (Table 1). The protein CsMADS_AGL72 was only 85 amino acids long while CsDL4 was 1633 amino acids long. All the proteins were unstable in nature with instability index more than 40 except CsCO, CsFLD, CsLFY, CsMADS_AGL82, and CsVIP3 which were stable with instability index 34.13, 88.86, 75.58, 92.14, and 27.29, respectively. This could be attributed to the presence of high level of α-helices in their tertiary structures (Fig. 5) except CsVIP3. The proteins CsSPB, CsSPL1 and CsSPL2 had similar structures despite having dissimilar length. The proteins CsBFT and CsTFL despite having the same length (173 aa) had different molecular weights i.e., 19234.97 Da and 19388.09 Da, respectively. This could be attributed to the variation in their amino acid composition (Table 2). However, both the genes showed great variation in genomic organization (Fig. 1b) and conserved motifs (Fig. 2a). The promoter region of CsBFT had additional CREs GT1CONSENSUS, and PYRIMIDINE BOX besides the DOFCOREZM present CsTFL (Additional file 1: Table S2). However, CsBFT lacked the TATA box present in CsTFL.

**Table 1:** Physical and chemical properties of the flowering related proteins

Protein name	Gene ID	Length (aa)	Molecular weight (Da)	pI	Instability index	Stability	Aliphatic index	GRAVY index	pFam domain	Subcellular location	Remarks (Suggested biotechnological approach for inducing precocious flowering)
CsFT	Cs7g07420.1	207	23143.33	6.29	47.17	Unstable	70.14	-0.928	Rdx family	Extracellular	Ectopic expression of an *FT* homolog conferred an early flowering phenotype in trifoliate orange [75]
CsCO	Cs5g20660.3	437	49268.88	6.87	34.13	Stable	87.92	-0.216	Methylenetetrahydrofolate reductase	Extracellular	*CO* overexpression resulted in early phenotype in *Arabidopsis* [76]
CsSOC1	Cs3g20780.1	1065	118360.4	5.23	45.83	Unstable	75.73	-0.605	KOW motif	Nuclear	Overexpression of *SOC1* suppressed the late flowering phenotype in *Arabidopsis* [77]
CsFLD	Cs7g24530.1	722	79152.14	9.35	34.26	Stable	88.86	-0.191	Flavin containing amine oxidoreductase	Nuclear	Overexpression of *FLD* induced early flowering in *Brassica juncea* [78]
CsLFY	Cs7g19530.1	398	44568.31	7.64	37.11	Stable	75.58	-0.569	DNA Binding Domain (C-terminal) Leafy/Floricaula	Nuclear	Constitutively expression of *LFY* promoteed flower initiation in *Arabidopsis* [79]
CsSVP	Cs1g20360.1	217	24646.96	6.18	56.63	Unstable	87.19	-0.69	SRF-type transcription factor (DNA-binding and dimerisation domain)	Nuclear	*svp* mutants passed more rapidly through the vegetative developmental stages in *Arabidopsis* [80]
CsTFL	Cs6g15000.1	173	19388.09	7.96	48.61	Unstable	78.27	-0.25	Phosphatidylethanolamine-binding protein	Cytoplasmic	*tfl1-1* mutation caused early flowering in *Arabidopsis* [81]
CsBFT	Cs9g16990.1	173	19234.97	9.16	40.78	Unstable	78.84	-0.305	Phosphatidylethanolamine-binding protein	Cytoplasmic	*BFT* over-expression caused late flowering in *Arabidopsis* [82]
CsMADS_AGL61	Cs2g21850.4	433	49222.5	9.26	50.33	Unstable	72.29	-0.897	Zinc finger C-x8-C-x5-C-x3-H type	Extracellular	*MADS_AGL* genes regulate flowering via various regulatory pathways [83, 84]
CsMADS_AGL82	Cs6g13050.1	485	55194.13	9.16	38.26	Stable	92.14	-0.247	PPR repeat	Mitochondrial	
CsMADS_AGL31	Cs1g26410.2	519	59,579	4.55	52.02	Unstable	85.24	-0.32	Cyclin, N-terminal domain	Extracellular	
CsMADS_AGL3	Cs5g12270.1	175	20207.84	5.85	55.62	Unstable	85.77	-0.592	K-box region	Nuclear	
CsMADS_AGL70	Cs2g04260.3	447	51135.08	6.66	41.4	Unstable	94.88	-0.327	Protein tyrosine and serine/threonine kinase	Extracellular	
CsMADS_AGL72	Cs9g16575.1	85	9753.47	9.51	42.6	Unstable	75.65	-0.146	SRF-type transcription factor (DNA-binding and dimerisation domain)	Nuclear	
CsMADS_AGL35	Cs6g01880.1	237	27186.28	9.37	56.77	Unstable	67.47	-0.713	SRF-type transcription factor (DNA-binding and dimerisation domain)	Nuclear	
CsTSF	Cs6g16420.1	146	16815.81	10.16	68.02	Unstable	76.1	-0.638	None	Membrane bound chloroplast	*TSF* overexpression caused a precocious flowering phenotype independent of photoperiods in *Arabidopsis* [85]
CsAP3	Cs1g24860.1	223	25761.15	9.39	44.23	Unstable	69.1	-0.856	SRF-type transcription factor (DNA-binding and dimerisation domain)	Nuclear	*AP3* negatively regulates *BANQUO* genes whose expression promotes early flowering [86]
CsAP2	Cs8g17390.1	510	55237.62	6.06	51.41	Unstable	56.08	-0.676	AP2 domain	Nuclear	Over-expression of miRNA targeted *AP2* and promotes early flowering [87]
CsWUS	Cs8g17610.1	208	24103.7	9.33	68.12	Unstable	60.48	-0.756	Homeodomain	Nuclear	Overexpression of *OsWOX13* (*WUS* Homeobox Transcription Factor) in rice resulted in early flowering [88]
CsCRY1	Cs3g18240.5	502	56830.21	5.33	55.05	Unstable	71.33	-0.594	FAD binding domain of DNA photolyase	Nuclear	Allele carrying the gain-of-function *CRY2-Cvi* flowers much earlier in *Arabidopsis* [89]
CsCRY2	Cs9g10510.2	644	73691.23	5.49	46.07	Unstable	77.55	-0.487	FAD binding domain of DNA photolyase	Nuclear	*OsiCRY2 Arabidopsis* over-expressers exhibited early flowering by 10–15 days in rice [90]
CsDL4	Cs4g01340.1	1633	183255.6	6.47	45.46	Unstable	93.04	-0.163	Ribonuclease III domain	Nuclear	Mutations of *DL* cause complete homeotic transformation of carpels into stamens [91]
CsGI	Cs3g21790.7	941	103301.3	6.19	57.18	Unstable	93.72	-0.05	None	Nuclear	Overexpression of *GI* promoted early flowering in *Arabidopsis* [92]
CsPHYB	Cs9g02220.2	1090	120896.4	5.84	44.14	Unstable	94.2	-0.108	Phytochrome region	Extracellular	Loss of function of *GmPHYA2/E4* or *GmPHYA3/E3* significantly shortened the time to flowering in soybean [93]
CsFLK	Cs8g13220.1	521	56104.4	4.66	56.22	Unstable	70.33	-0.565	KH domain	Extracellular	Mutant having inactivated *FLK* via T-DNA insertion exhibited late flowering phenotype [94]
CsSEP2	Cs6g19680.1	243	27894.81	9.01	41.84	Unstable	83.09	-0.674	K-box region	Nuclear	Overexpression of *SEP3* causes similar phenotypes as 35 S:AP1 to promote the flowering of *Arabidopsis* [95]
CsPI	Cs4g06140.3	238	27357.43	8.15	50.63	Unstable	92.14	-0.445	SRF-type transcription factor (DNA-binding and dimerisation domain)	Nuclear	Mutations in *PI* resulted in the homeotic transformation of petals to sepals and stamens to carpels [96]
CsMAF1	Cs2g14610.2	222	24322.14	9.65	46.95	Unstable	94.91	0.089	Cofactor assembly of complex C subunit B	Extracellular	*MAF2* overexpression between generations delays flowering in *Arabidopsis* [61]
CsSHP1	Cs7g16960.1	127	14425.72	10.01	57.53	Unstable	82.99	-0.547	SRF-type transcription factor (DNA-binding and dimerisation domain)	Nuclear	*shp1 shp2* double mutant fruits do not open at maturity in *Arabidopsis* [97]
CsCEN	Cs8g15080.1	172	19665.50	8.92	45.62	Unstable	76.40	-0.357	Phosphatidylethanolamine-binding protein	Cytoplasmic	Silencing *GoCEN* led to early flowering in cotton [98]
CsFLC	Cs7g07200.1	200	22395.88	9.40	41.16	Unstable	98.60	-0.308	-	Nuclear	Loss-of-function of *FLC* promoted flowering in *Arabidopsis* [99]
CsEMF1	Cs4g24260.1	1247	137747.91	7.17	52.08	Unstable	61.57	-0.755	Protein EMBRYONIC FLOWER 1	Extracellular	Loss-of-function mutations in *Arabidopsis EMF* produced a single terminal flower on all nodes [69]
CsFRI	Cs7g14090.1	618	68196.92	8.63	48.26	Unstable	83.82	-0.361	Frigida-like	Nuclear	Early-flowering *Arabidopsis* ecotypes have loss of *FRI* function with alleles containing one of two different deletions that disrupt the open reading frame [66]
CsTEM1	Cs7g09120.1	377	41911.27	8.89	44.87	Unstable	67.48	-0.640	B3 domain-containing transcription factor LEC2-like	Extracellular	*tem1-1* mutant enhanced the early flowering phenotype in *Arabidospsis* [100]
CsSPB	Cs2g19530.1	189	21086.62	9.02	52.39	Unstable	49.58	-1.110	Squamosa promoter binding-like protein	Nuclear	Over-expression of *SBP* resulted in delayed flowering in *Arabidopsis* [101]
CsSPL1	Cs9g00360.2	1038	115346.83	8.31	53.05	Unstable	81.40	-0.389	Squamosa promoter binding-like protein	Nuclear	Ectopic expression of *RcSPL1* in *Arabidopsis* accelerated the vegetative phase transition and flowering [102]
CsSPL2	Cs7g07070.8	480	52509.43	7.17	49.36	Unstable	58.35	-0.678	Squamosa promoter binding-like protein	Nuclear	*Spl2* contribute to both juvenile-to-adult vegetative transition and the vegetative-to-reproductive transition in *Arabidopsis* [103]
CsSUF4	Cs2g13420.2	402	44137.22	7.66	63.33	Unstable	68.31	-0.284	-	Nuclear	*suf4* mutant exhibited an earlier flowering phenotype in *Arabidopsis* [104]
CsVIN3	Cs7g01480.1	1211	134654.43	5.56	55.08	Unstable	61.11	-0.828	Protein OBERON	Nuclear	*Vin3* mutants completely block vernalization response in *Arabidopsis* [72]
CsVIP3	Cs4g26180.2	315	34003.42	5.66	27.29	Stable	83.56	-0.063	-	Cytoplasmic	*vip3* mutant plants flowered earlier than *flc* mutants in *Arabidopsis* [105]
CsDELLA	Cs2g10860.1	594	65010.28	5.07	50.32	Unstable	78.10	-0.252	Transcription factor GRAS	Nuclear	Loss of individual *DELLA* genes resulted in only a minor hastening in flowering in *Arabidopsis* [106]
CsZTL	Cs4g02700.1	616	67568.75	5.38	43.76	Unstable	83.78	-0.168	-	Cytoplasmic	Over expression of *ZTL* causes late flowering phenotype in *Arabidopsis* [107]
CsFT3	Cs7g24270.1	772	88200.25	9.13	42.98	Unstable	93.76	-0.200	QUIRKY-like	Extracellular	*CcFT3* overexpression induced precocious flowering in many transgenic lines in Carrizo [108]

**Table 2:** Proteins modelled using Phyre2 and percentage composition of essential amino acids

Protein name	Template	PDB header/ fold	Essential amino acid composition (%)								
			His	Ile	Leu	Lys	Met	Phe	Thr	Trp	Val
CsFT	c2obkE	structural genomics	1.0	2.9	5.3	15.0	1.9	2.4	4.8	0.0	10.1
CsCO	c6fcxA	oxidoreductase	2.7	8.0	8.5	6.6	3.4	4.6	5.7	1.4	5.9
CaSOC1	c5oikZ	Transcription	2.0	5.4	6.3	6.0	2.9	2.4	4.5	0.8	8.5
CsFLD	c2xagA	Transcription	2.1	4.2	11.4	6.9	2.5	4.2	4.4	1.0	7.2
CsLFY	c2vy2A	Transcription	2.8	3.0	8.3	5.8	1.5	3.0	2.8	1.5	6.8
CsSVP	d1n6ja	SRF-like	1.8	5.5	11.5	8.8	2.3	2.3	2.3	1.8	5.1
CsTFL	d1qoua	Ribosome	2.9	5.2	5.8	2.3	2.9	6.4	7.5	0.6	9.8
CsBFT	d1qoua	PEBP-like	2.9	5.8	4.6	3.5	3.5	4.6	6.9	0.6	10.4
CsMADS_AGL61	c6yvuB	cell cycle	3.5	4.8	8.3	9.2	1.2	2.5	4.2	1.2	5.3
CsMADS_AGL82	c4m57A	rna binding protein	1.2	6.2	11.1	8.7	4.3	2.1	4.3	1.4	5.6
CsMADS_AGL31	c1h28B	cell cycle/ transferase substrate	1.9	6.7	8.1	6.7	3.9	5.2	2.9	1.0	7.5
CsMADS_AGL3	c4ox0D	Transcription	3.4	3.4	13.1	4.6	2.3	2.3	5.1	1.1	5.7
CsMADS_AGL70	c3cblA	Transferase	2.7	6.5	10.7	7.6	2.0	4.5	4.3	1.1	7.2
CsMADS_AGL72	c7nb0A	plant protein	0.0	7.1	5.9	10.6	3.5	8.2	4.7	0.0	7.1
CsMADS_AGL35	d1n6ja	SRF-like	1.3	3.8	9.3	11.0	5.9	3.8	3.8	1.7	4.2
CsTSF	c5mmi6	Ribosome	3.4	4.8	11.0	13.7	5.5	3.4	1.4	0.7	4.1
CsAP3	d1mnma	SRF-like	2.7	4.5	9.0	9.4	3.6	3.1	7.6	0.4	4.0
CsAP2	c7et4G	dna binding protein/dna	1.8	2.5	5.7	4.5	1.8	4.5	5.5	1.4	5.5
CsWUS	c6wigA	plant protein/dna	5.3	3.4	8.7	6.7	5.3	3.4	6.2	1.9	3.4
CsCRY1	c1u3cA	signaling protein	2.4	3.8	8.2	2.8	1.8	4.0	4.4	3.2	5.8
CsCRY2	c6k8kA	signaling protein	2.4	4.0	10.4	5.3	1.9	4.0	3.9	3.7	5.1
CsDL4	c7eldA	Hydrolase	2.8	5.9	11.8	6.7	1.8	4.8	3.4	0.7	6.1
CsGI	c7wa4A	circadian clock protein	3.3	5.8	11.2	3.9	1.6	3.2	4.0	1.7	5.4
CsPHYB	c7rzwA	gene regulation	3.1	6.1	10.1	5.0	2.4	3.8	5.1	0.9	7.9
CsFLK	c2anrA	rna-binding protein/rna	2.9	5.0	4.6	3.5	3.3	1.3	3.8	0.6	8.8
CsSEP2	c7nb0A	plant protein	1.2	3.7	14.0	7.4	2.1	2.5	5.3	0.8	3.3
CsPI	c7nb0A	plant protein	2.5	7.1	11.8	8.4	2.1	4.2	5.0	0.4	5.0
CsMAF1	d1ijdA	Light-harvesting complex subunits	0.9	5.4	11.7	5.9	1.4	5.4	7.2	1.8	6.8
CsSHP1	d1n6jA	SRF-like	0.0	6.3	11.0	11.0	1.6	2.4	4.7	0.8	3.1
CsCEN	d1qouA	PEBP-like	2.3	4.7	5.8	4.7	3.5	7.0	7.0	0.6	10.5
CsFLC	c7nb0A	Plant protein	1.0	4.5	15.0	7.5	2.0	2.5	5.0	0.5	4.5
CsEMF1	c4mc5C	Viral protein	4.0	4.3	6.9	7.7	2.5	3.5	5.1	0.8	4.2
CsFRI	c5ch6B	Transcription	2.6	6.6	8.1	8.3	2.4	3.6	4.4	1.0	6.3
CsTEM1	d1widA	DNA-binding pseudobarrel domain	1.9	4.2	6.6	8.0	2.7	3.7	4.5	1.6	6.6
CsSPB	d1ul4A	SBT domain	3.2	2.1	5.3	11.6	2.1	2.6	3.2	0.0	4.8
CsSPL1	d1ul4A	SBT domain	2.7	3.9	10.1	5.8	1.9	3.9	3.7	1.3	7.3
CsSPL2	d1ul4A	SBT domain	2.5	2.7	7.5	6.7	2.5	3.8	5.6	1.5	4.2
CsSUF4	c6j0daA	Transcription	3.0	3.5	4.7	5.0	3.0	4.2	4.5	1.5	10.2
CsVIN3	c6lthO	Gene regulation	2.2	3.2	7.3	7.3	2.6	3.8	3.1	0.8	5.1
CsVIP3	c2ymuA	Unknown function	3.2	3.2	8.3	4.1	2.9	3.2	7.6	3.2	10.2
CsDELLA	c6kpdC	Transcription	2.7	3.7	9.3	3.9	3.2	4.4	4.7	1.2	5.7
CsZTL	c5svuD	Circadian clock protein	2.1	3.7	10.6	2.4	1.6	4.5	5.7	2.6	8.3
CsFT3	c7t7vA	Exocytosis	3.1	6.1	10.4	6.1	2.6	4.1	5.4	2.2	8.4

The pFam domain analysis revealed that most of the proteins belonged to SRF-type transcription factor (DNA-binding and dimerization domain) family and squamosa promoter binding-like protein (Table 2). The rest of the proteins belonged to Phosphatidylethanolamine-binding protein family, K-box region and Rdx family. The determination of subcellular location of these proteins gave insights into the place of action to the proteins. Out of the total, seventeen proteins were located in nucleus and rest were located either in the cytoplasm or secreted as extracellular proteins.

The 3D-structures of the proteins were determined *via* homology and analogy modelling using Phyre2 web portal. The structures created with 100% confidence are shown in Fig. 5. The proteins CsCO, CsCRY1, CsLFY, CsDL4, CsMADS_AGL31 and CsMADS_AGL82 were composed of α-helices only. The proteins CsBFT, CsFLD, CsAP2, CsCRY2, CsMADS_AGL35, CsPHYB, CsSOC1, CsSHP1, CsTFL, CsCEN, CsTEM1, CsSPB, CsSPL1, CsSPL2, CsVIP3, CsDELLA, CsFT3, and CsZTL had β-sheets in addition to α-helix. The proteins CsBFT and CsTFL, and CsSPB, CsSPL1 and CsSPL2 had the similar structures. The α-helix appeared to be the dominant structure which is known to represent 30% of the structure of globular proteins [109]. A β-sheet is more flat, thin and flexible as compared to an α-helix [110]. However, α-helix motifs possess higher stability than β-sheets [111]. Thus, the presence of high number of α-helices accounted for the stability of proteins CsCO, CsFLD, CsLFY and CsMADS_AGL82 (Table 1). The templates used for the prediction of the structure and their PDB header along with the composition of essential amino acids are given in Table 2. The templates were mostly the proteins involved in transcription, nucleic acid binding, SBT domain and were SRF-like proteins. Leucine was the most abundant essential amino acid present in the proteins and tryptophan was the least abundant. The leucine rich repeats form a conformation which increases the surface area, thereby, mediating protein-protein interactions [112].

**Fig. 6:**
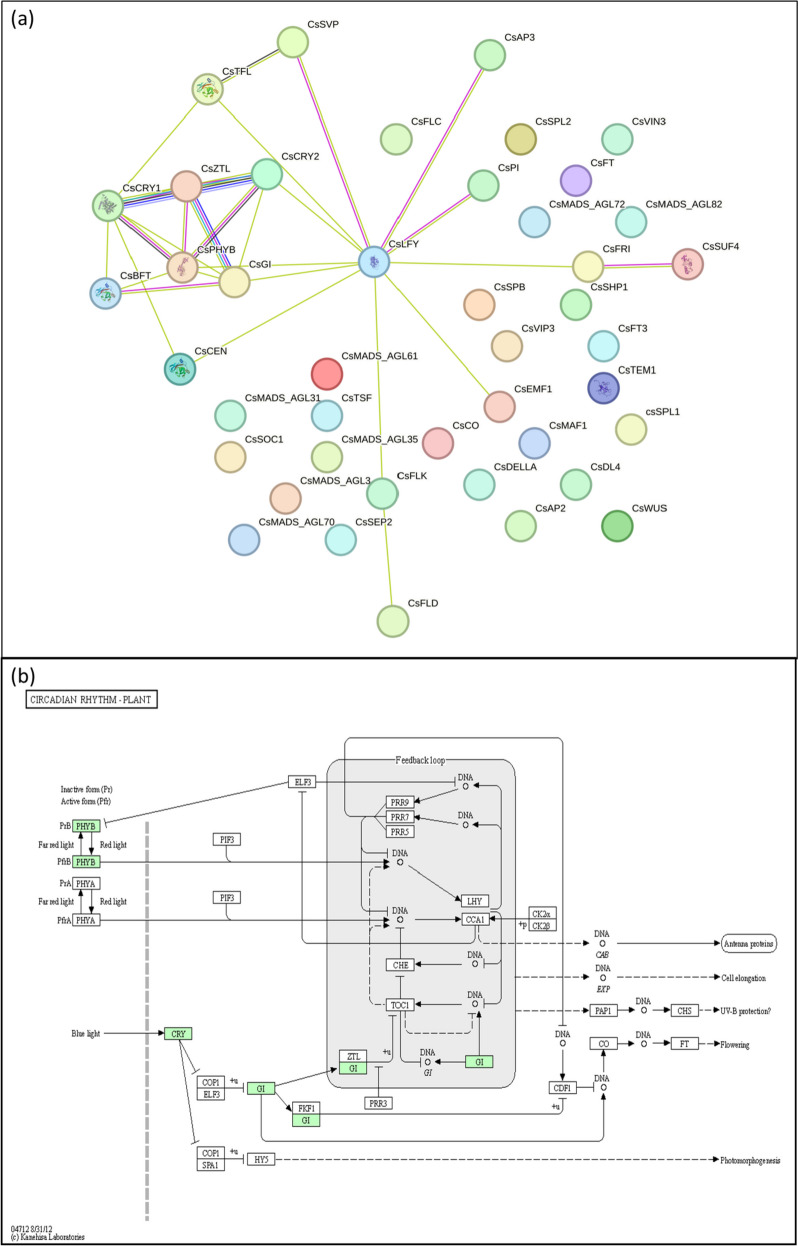
In-silico studies of the protein sequences of flowering genes (**a**) Protein-protein interaction network of flowering related genes in sweet orange. **b** Proteins involved in circardian rhythm pathway

The protein-protein interaction network is shown in Fig. 6a. The network had 43 nodes and 29 edges; average node degree of 1.35. Majority of the interactions were either text mined (green edges) or experimentally determined (pink edges). The proteins had more interactions among themselves than what would be expected for a random set of proteins of the same size and degree distribution drawn from the genome. Such enrichment indicated that the proteins are at least partially biologically connected, as a group. The string clustering of the proteins is given in Additional file 1: Table S5. The proteins were clustered into 11 clusters; which included MADS MEF2-like and PEBP binding proteins. Based on k-means clustering, the proteins were clustered into five groups as shown by five colors in Fig. 6a and Additional file 1: Table S6. The KEGG pathway analysis showed the involvement of four proteins (PHYB, CRY, GI, and ZTL) in circadian rhythm pathway which ultimately control the expression of *CO* and *FT* which regulate flowering (Fig. 6b).

### Comparative genomics

The comparative genomics was carried out to analyse the evolutionary history of the flowering genes in citrus species. The genome databases of five citrus species were compared viz., sweet orange, clementine, mandarin, citron, and pummelo (Table 3). Citron has the largest genome followed by pummelo. Sweet orange had the lowest GC content of all four species. Despite having comparatively smaller genome, sweet orange had the greatest number of pathways characterized including metabolic reactions, transport reactions and chemical compounds. The GO annotation showed that the maximum number of genes had been annotated in citron followed by pummelo. Sweet orange had the least number of genes annotated according to citrus genome database.

**Table 3:** Comparative of genomic databases of four species of citrus

	Clementina	Pummelo	Citron	Sweet orange
**Database**				
Genome Size (bp)	290201842.00	345779982.00	406057947.00	327944670.00
Genes	24,528	42,872	47,399	24,727
Genes of known or predicted molecular function	9,130	12,598	13,693	10,500
%GC Content	33.6	34.9	32	31.28
Pathways	484	265	254	583
Metabolic Reactions	2,776	1,665	1,639	3,749
Transport Reactions	26	19	18	40
Compounds	2,310	1,491	1,465	3,079
**Gene Ontology**				
GO Annotations	13,552	24,620	26,942	12,074
Biological process	8,300	14,113	15,128	6,702
Cellular component	5,125	9,685	10,429	4,222
Metabolic function	5,184	9,223	9,576	4,367

The structures of flowering genes were compared within five citrus species sweet orange, clementine, mandarin, citron, and pummelo. The proteins sequences which shared alignments are shown in Table 4. The alignments did not follow a particular pattern for example *WUS* gene was similar in pummelo and citron, while *FLK* gene was similar in pummelo and sweet orange. No gene was observed to be similar in all the five species. The only gene which was similar in four species (except citron) was *LFY*. The genes *TFL1, BFT* and *SVP* were similar in three species: clementine-pummelo-sweet orange; clementine-citron-sweet orange; and pummelo-citron-sweet orange respectively. The gene *SVP* also showed similarity between clementine and mandarin.

**Table 4:** Pairs of protein sequences showing 100% alignment analysed via Clustal Omega

S. No.	Proteins with 100% alignment
1	CgWUS-CmWUS
2	CgFLK-CsFLK
3	CmAP3-CmPI
4	CcSOC1-CrSOC1
5	CcLFY-CgLFY-CrLFY-CsLFY
6	CcFLD-CsFLD
7	CcAP3-CsAP3
8	CgAP3-CrAP3
9	CcTFL1-CgTFL1-CmTFL1
10	CcBFT-CrBFT-CsBFT
11	CgSVP-CmSVP-CsSVP
12	CcSVP-CrSVP
13	CcSEP2-CgSEP2
14	CmSEP2-CsSEP2

**Fig. 7:**
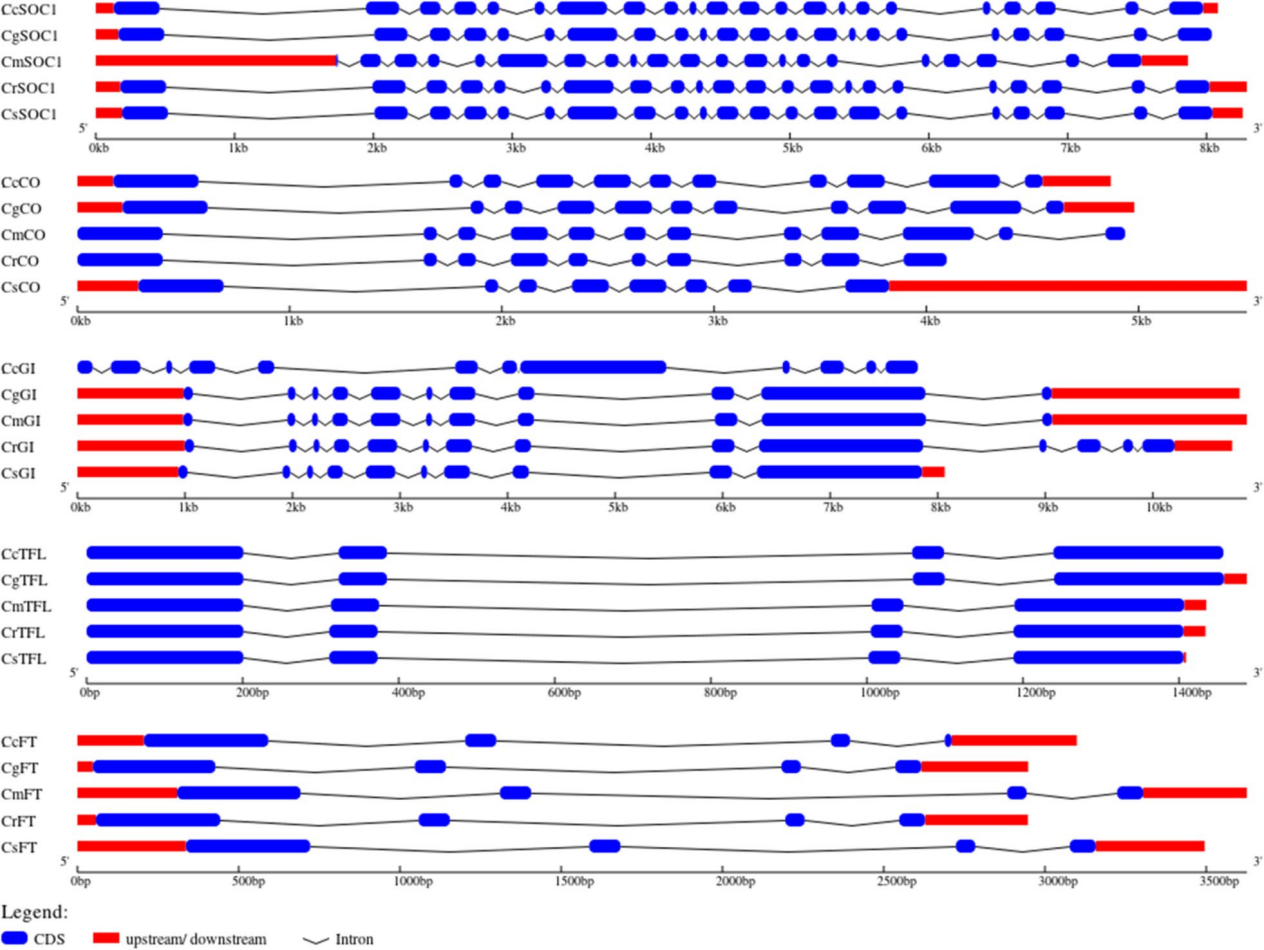
Variations in the genomic organization of genes *SOC1 CO, GI, TFL* and *FT* in different citrus species (clementine, pummelo, citron, mandarin and sweet orange denoted as Cc, Cg, Cm, Cr and Cs respectively)

The variation in genomic structure of five genes viz., *CO, SOC, TFL, GI* and *FT* were studied in five species. These genes showed variation within the citrus species are shown in Fig. 7. Despite having similar function and common genera, dissimilarities in intron-exon organization of these genes were observed. The gene *SOC1* had similar structure in Cc, Cr and Cs except for the length of the distal downstream region while *CgSOC1* sequence lacked the same. While in case of *CmSOC1* a highly elongated upstream element was present. Similarly, in case of *CO, GI, TFL* and *FT*, presence and absence of upstream/ downstream elements and differences in lengths of introns resulted in variations among the genes.

**Fig. 8:**
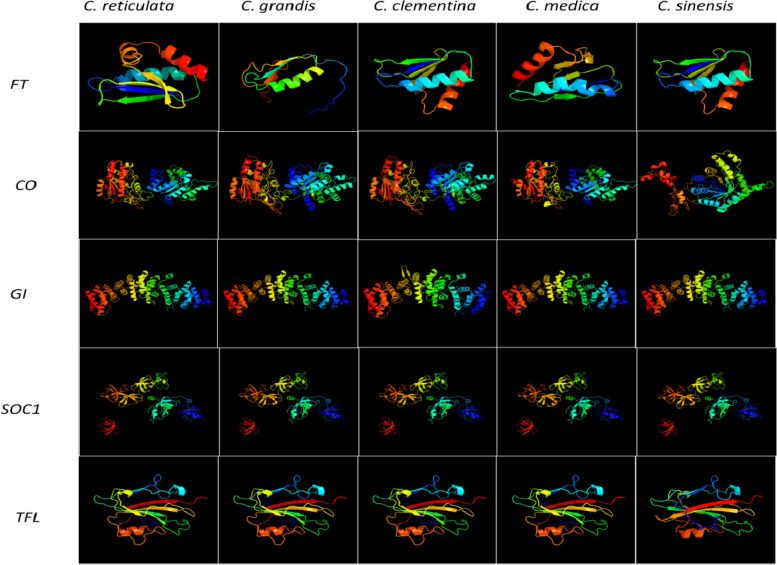
Protein structures of genes with variation in genomic organization

The protein structures of the genes showing variations are given in Fig. 8. The genes showing similar gene structure but different protein structure would indicate changes in post-transcriptional modifications and genes showing dissimilar gene structure but similar protein structure would indicate variation in non-coding sequences and/or un-translated regions in the genes. The genes *GI, SOC1* and *TFL* despite having dissimilar genomic structures; their proteins had similar tertiary structures. This means that the genes had different intronic sequences. The protein FT had different structure in all the species. Similarly, the CO protein had similar structure in all the species except sweet orange.

**Fig. 9:**
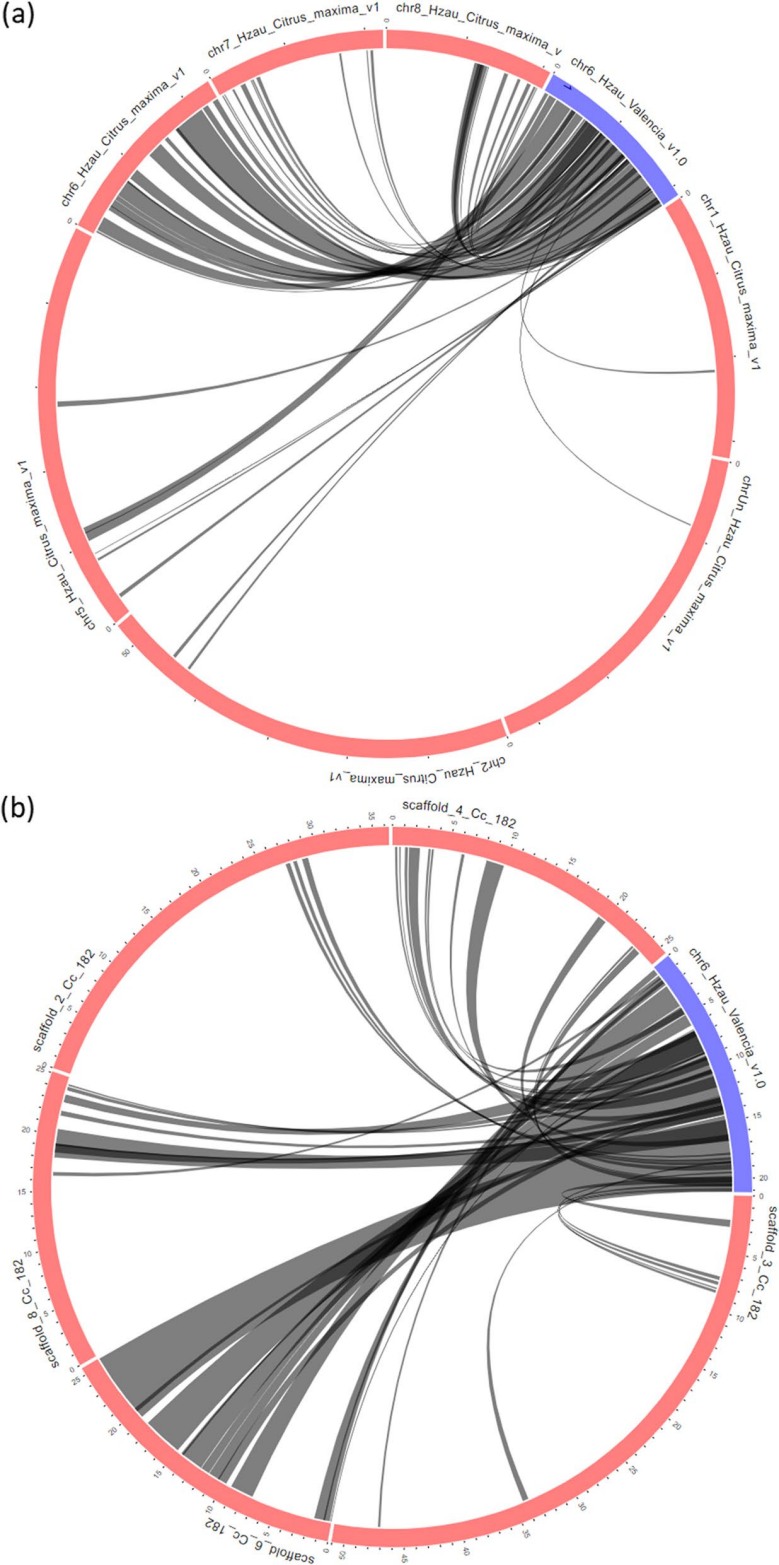
Syntenic blocks of chromosome 6 of sweet orange with (**a**) *C. maxima* genome and (**b**) clementine genome

Synteny (collinearilty) analysis helps in identification of homologous genes and gene order between genomes of different species [113]. Synteny blocks offer an alternative and more practical approach for comparative genomics which is dependent on the identification of homologs [114, 115]. It was first described as homologous genetic loci that co-occur on the same chromosome [116, 117]. A more formal definition is the regions of chromosomes among genomes sharing a common order of homologous genes which are derived from a common ancestor [118]. In case of flowering gene, the maximum number of genes were present on chromosome 6 in sweet orange (Fig. 1a). Therefore, the comparative analysis was carried out for genes present on chromosome 6 of sweet orange with genome of *C. maxima* and clementine. The synteny blocks are shown in Fig. 9. A total of 43 syntenic blocks were observed between on chromosome 6 of sweet orange and *C. maxima* (Fig. 9a). Majority of genes were collinear with chromosome 6 of sweet orange showing the conserved nature of genes during evolutionary progress. Similarly, in case of clementine, 41 syntenic blocks were observed (Fig. 9b). Majority of genes were located on scaffold 6.

### Expression analysis of flowering genes

**Fig. 10:**
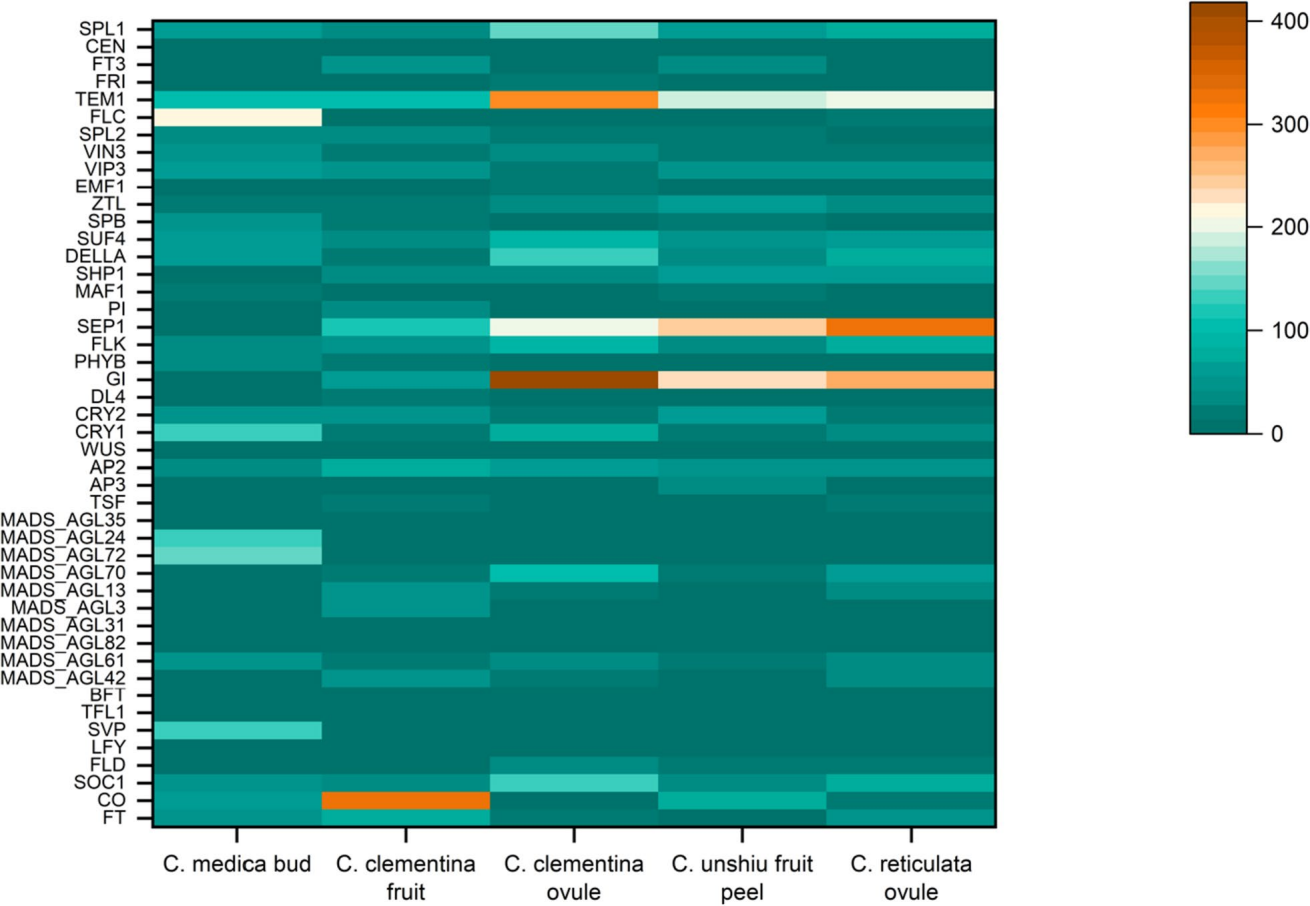
Heatmap of expression analysis of flowering genes in different tissues of citron, clementine, *C. unshiu* and mandarin

A heatmap was constructed for flowering genes in various tissues of *Citrus* species. The flowering genes are expressed in shoot apical meristem and floral buds. The rkpm values of the expression data are given in Additional file 1: Table S7. The expression of *FT* gene was the maximum in fruit (clementine) followed by that in ovule (mandarin) (Fig. 10). Similarly, the expression of *CO* gene was observed to be the maximum in mandarin fruit. Incomplete expression data were observed for certain genes such as *TFL1*, *MADS_AGL82, SVP, MADS_AGL72, MADS_AGL24, MADS_AGL35* and *WUS.* The expression of gene *GI* was the highest of all which was observed in clementine ovule. The same tissue also observed the highest expression for genes *SOC1, FLD, MADS_AGL70*, *FLK, DELLA, SUF4, EMF1, TEM1, FRI, SPL1*. Similarly, mandarin ovule observed the highest gene expression for genes *TSF* and *SEP1* and buds of *C. medica* had highest gene expression for *LFY, SVP, MADS_AGL61, MADS_AGL72, MADS_AGL24, CRY1, PHYB*, *MAF1, SPB, VIP3, VIN3*, and *FLC.* The findings suggested that most of the flowering genes are expressed in bud and ovules.

### qRT-PCR analysis of flowering genes in different tissues of sweet orange and comparison with other species

**Fig. 11:**
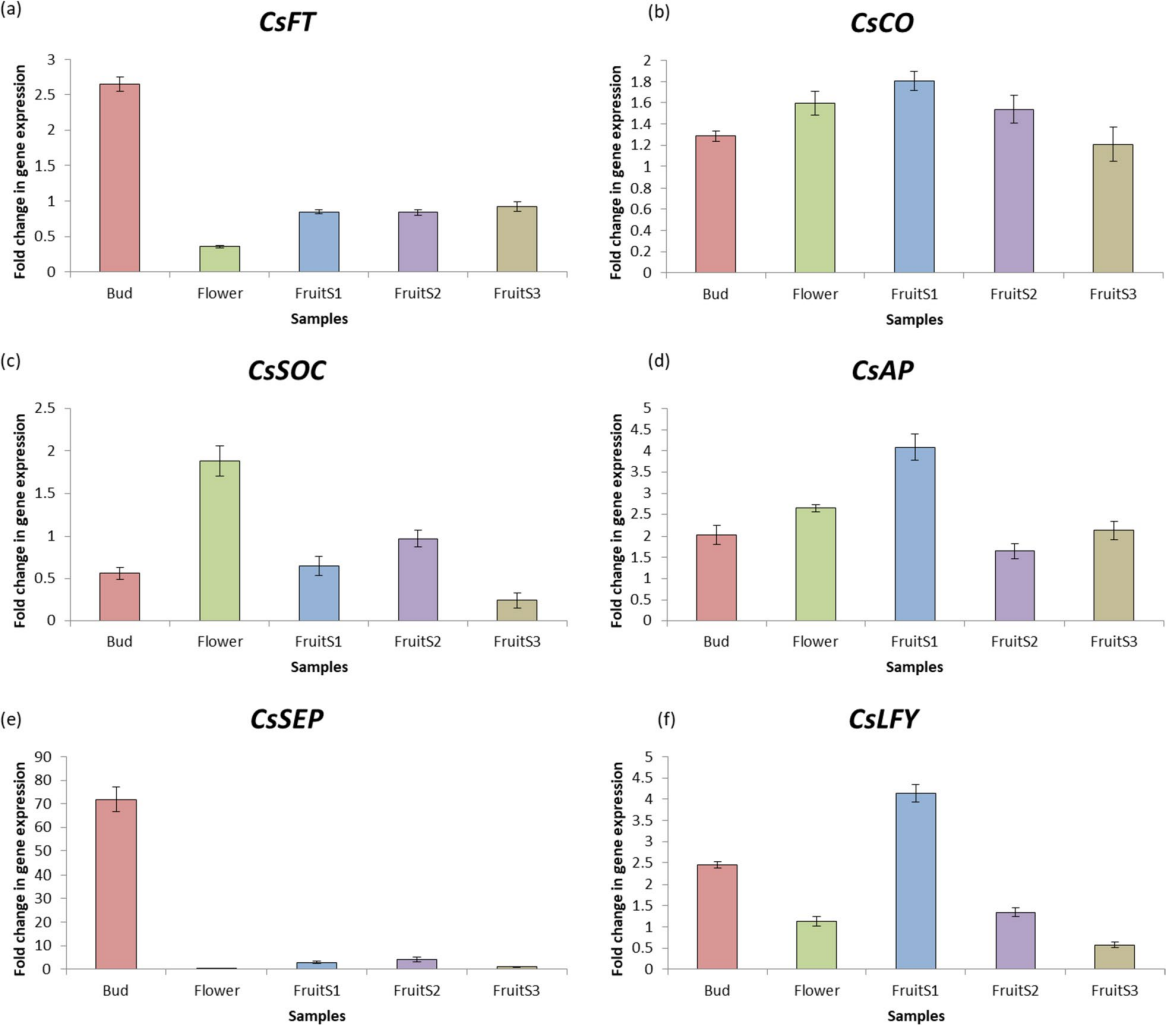
Fold change in gene expression of flowering genes in various tissues of sweet orange (**a**) *CsFT* (**b**) *CsCO* (**c**) *CsSOC* (**d**) *CsAP* (**e**) *CsSEP* (**f**) *CsLFY*

The expression analysis of six flowering related genes (*CsFT*, *CsCO*, *CsSOC*, *CsAP*, *CsSEP* and *CsLFY*) was determined in various tissues of sweet orange (Fig. 11). The leaf sample was taken as control and gene expression was compared with flower bud, fully grown flower and three stages of fruits (FruitS1, FruitS2 and FruitS3). The melt curves of the genes are shown in Additional file 1: Fig. S1. The results indicated that the maximum expression of *CsFT* was observed in bud stage and the minimum expression was observed in fully grown flower stage (Fig. 11a). The *CsFT* expression increased with increasing fruit development stage. Contrarily, the expression of *CsCO* decreased with increasing fruit development stage (Fig. 11b). The *CsCO* expression was higher in flower as compared to bud stage. *CsSOC* showed the maximum expression in flower stage (Fig. 11c) while *CsSEP* showed the maximum expression in bud stage (Fig. 11e). The genes *CsAP* and *CsLFY* showed the maximum expression in early fruit development stage (FruitS1: 7–8 days after flowering) as shown in Fig. 11d and f. The results showed that the bud, fully mature flower and early fruit developmental stage (Fruit S1) could be used for targeting the expression of flower genes.

**Fig. 12:**
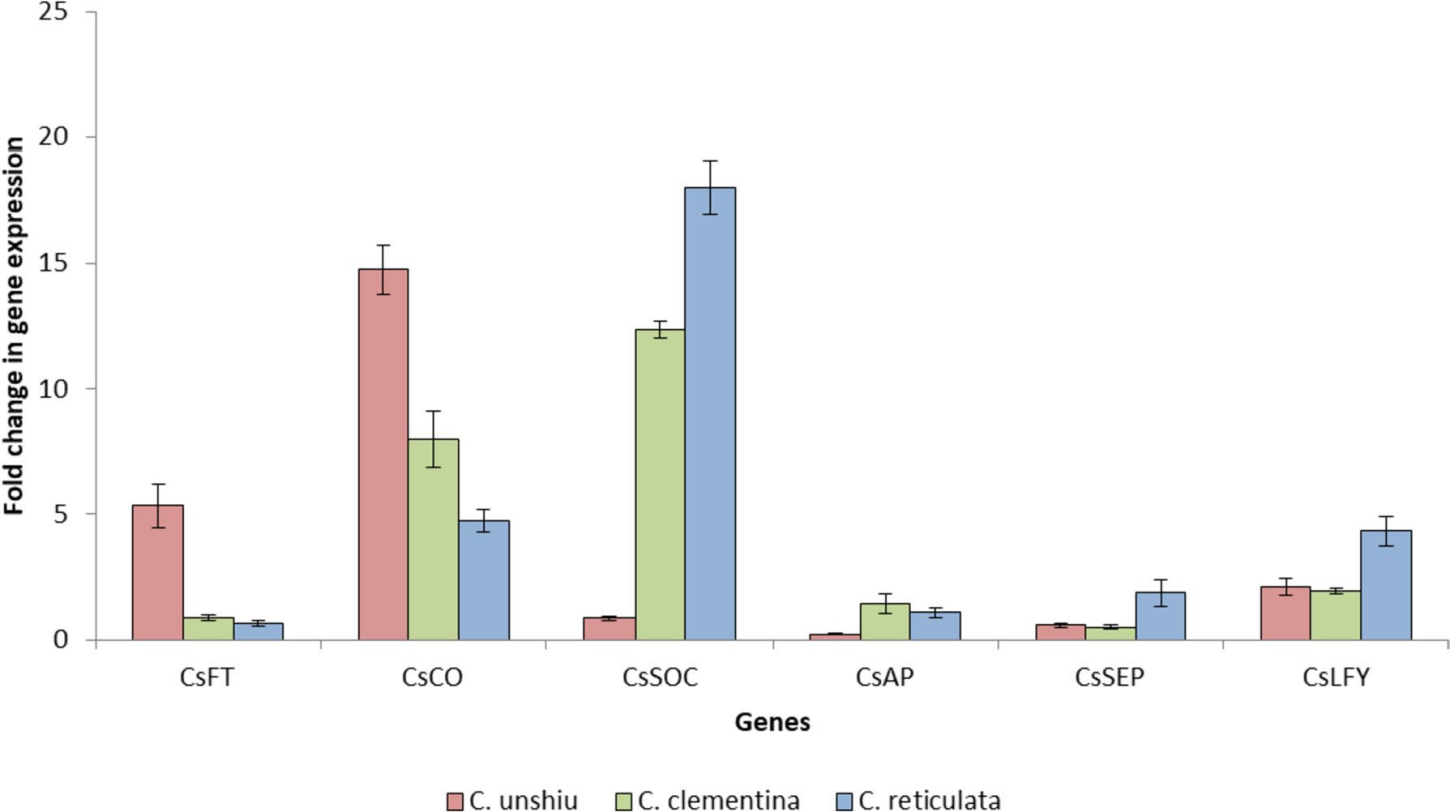
Fold change in expression of flowering genes in leaf tissues of *C. unshiu*, *C. clementina* and *C. reticulata* using *C. sinensis* as control

The leaf samples were used to determine the expression of flowering genes in various citrus species (*C. sinensis, C. unshiu, C. clementina* and *C. reticulata*). The *C. sinensis* leaf tissue was taken as control to compare the expression of levels of *CsFT*, *CsCO*, *CsSOC*, *CsAP*, *CsSEP* and *CsLFY* with other species (Fig. 12). The results indicated that the maximum expression of *CsFT* and *CsCO* was observed in *C. unshiu* while the maximum expression of *CsSOC*, *CsSEP* and *CsLFY* was observed in *C. reticulata*. *C. clementina* leaf tissue showed the maximum expression of *CsAP*.

## Discussion

Most of the *Citrus* species possess characteristic feature of long juvenility period; therefore they do not bear flowers or fruits for many years. This hinders and delays the breeding approaches for generation of improved *Citrus* varieties and cultivars. In order to break the juvenility via biotechnological approaches, a detailed study of the genes involved in flowering is required. Flowering in *Citrus* is a complex mechanism regulated by various genetic and environmental factors. The present study was carried out to identify genes responsible for flowering in sweet orange. The bioinformatics analysis gave insight into the structural and functional analysis of the genes and proteins. Moreover, the structure of genes and proteins were compared within various *Citrus* species to recognize their structural and functional similarities.

The study involved identification of 43 flowering related genes in sweet orange genome distributed across 9 chromosomes (Fig. 1a). The analysis of promoter sequence detected various CREs in the sequences which included GT1CONSENSUS (GRWAAW), CARG box (CWWWWWWWWG), TATA box, DOFCOREZM (AAAG), CCAAT box, ABRELATERD1 box (ACGTG), GARE box (TAACAAR), MYBGAHV (TAACAAA), Pyrimidine box (CCTTTT / TTTTTTCC) and CARE box (CAACTC) (Fig. 2a). These boxes regulate the transcription of genes *via* various mechanisms. Under inductive day length conditions, the activation of transcription of gene *FT* is facilitated by *CO* [119]. The stability of CO protein is affected by light; hence long day conditions result in accumulation of sufficient CO proteins which induce expression of *FT* gene [9, 120, 121]. The transcriptional activation occurs as follows. The *CO* encodes a nuclear protein which contains two zinc binding B-boxes and a CCT domain (comprised of CONSTANS, CO-like, TIME OF CAB1) [122, 123]. However, CO alone cannot activate transcription. The CCT domain of CO interacts with Nuclear Factor Y (NF-Y) complex [124, 125] which in turn binds to DNA in the form of a heterotrimeric complex which recognizes CCAAT *cis-*elements [126, 127]. Previous studies have shown the role of NF-Y complex in controlling flowering and such complexes are located downstream of CO in the photoperiodic pathway in case of *Arabidopsis* [128–130]. Nuclear factor Y (NF-Y) is a ubiquitous CCAAT-box binding transcription factor which is composed of three subunits i.e., NF-YA, NF-YB and NF-YC [131, 132]. The NF-Y, particularly NF-YB subunits, has been identified as a flowering time regulator in plants [126].

The GT1CONSENSUS is the binding site of GT-1 transcription factor (trihelix family) which effects the salicylic acid inducible pathogenesis-related gene expression [133]. The DOFs are a set of plant specific transcription factor whose core binding site is DOFCOREZM [134]. The Dof proteins include Dof1, Dof2, Dof3, and PBF [135]. Dof1 regulates activities of *c4pepc, cyppdk, and pepcZm2A* promoters which are involved in carbon metabolism [135].

The MYB (myeloblastosis) transcription factors contain the MYB domain which helps in DNA binding [136]. MYB transcription factors are classified based on the number of repeats present in their sequences which can vary from 1 to 4 [137]. In plants, MYB transcription factors play a key role in plant development, secondary metabolism, hormone signal transduction, disease resistance and abiotic stress tolerance [138], root development [139] and flowering [140]. Some MYB transcription factors can also participate in light, low-temperature, and osmotic stress induction responses [140]. A MYB-related protein known as FE has been found to positively regulate the *FT* and *FTIP1* (FLOWERING LOCUS T INTERACTING PROTEIN) in *Arabidopsis* [17]. ABA-responsive elements (ABREs) are basic leucine zipper (bZIP)-type ABRE binding proteins (AREBs) that function in response to abscisic acid treatment [141]. The CREs can be categorized as light-responsive such as GT1CONSENSUS, stress-responsive GT1GMSCAM4 and CAATBOX, hormone-responsive such as ABRELATERD1, and transcription factor binding sites such as DOFCOREZM [142].

The KEGG pathway analysis showed the involvement of four proteins (PHYB, CRY, GI, and ZTL) in circadian rhythm pathway which ultimately control the expression of *CO* and *FT* which regulate flowering (Fig. 6b). Circadian rhythms are a type of biological rhythms which occur periodically which take ∼24 h to complete one cycle [143]. Circadian rhythms are known to regulate various plant functions including flowering [144]. The role of circadian clocks in flowering has been well studied in *Arabidopsis.* The circadian rhythms are carried out as three feedback loops known as morning, central and evening loops [145]. The gene *GI* forms an important component of the evening loop which activates ZTL protein and acts along with it to degrades TOC1 (TIMING OF CAB EXPRESSION) protein [146]. The *TOC1* gene belongs to family of Pseudo-Response Regulators (PRRs) and help in synchronizing the signal of light between *PHYB* and clock rhythms [147].

Flowering plants possess multiple photoreceptors which are categorized based on the wavelength spectra they absorb which spans from UV-B to far-red (280 to 750 nm). These light harvesting proteins have been characterized as phytochromes (PHYs), cryptochromes (CRYs), ZTL proteins, and the UV resistance locus 8 (UVR8) [148–150]. The KEGG analysis revealed involvement of PHYB, CRY and ZTL in circadian rhythms. These photoreceptors (PHYBs, CRYs and ZTL) perceive light signals upon illumination and mediate photomorphogenic growth, via various mechanisms such as inhibition of hypocotyl elongation, promotion of cotyledon expansion, and accumulation of anthocyaninn [93, 151, 152].

The PHYs are plant-specific photoreceptors which mediate photoperiodic flowering by absorbing red and far-red light [152]. They undertake two photoconvertible forms, inactive Pr form which absorbs red light (λmax = 660 nm) and the active Pfr form which absorbs far-red light (λmax = 730 nm) [153, 154]. Similarly, CRYs and ZTL are photoreceptors which absorb blue light. Studies have shown that CRY1, CRY2 and PHYA are required to initiate flowering and stabilize CO protein, while PHYB promotes delayed flowering and deprivation of CO [155]. However, the deprivation of CO is activated in night and repressed by the day *via* COP1 (CONSTITUTIVE PHOTOMORPHOGENIC 1) and SPA1 (SUPPRESSOR OF PHYTOCHROME A) respectively [156]. COP1 and SPA1 are ubiquitin ligases to which CO binds directly which in turn inhibit their property of CO degradation to promote CO gene expression at the ending of the long- day photoperiod [121]. Various studies have found that loss-of-function mutations in these genes result in delay of flowering under long days but have little or no effect under short days [157]. Similarly, GI plays significant role in red light signalling, regulation of circadian rhythms, and controlling flowering time [92]. Under day/night cycle, the GI controls the expression of CO such that CO mRNA is expressed in cases when plants are exposed to light under long days but not under short days [158]. The exposure to light is required for the activation of CO protein functioning [120]. It has been proposed the expression of FT is directly activated by CO in response to light, resulting in flowering [120]. ZTL is a circadian clock protein found in *Arabidopsis* which senses blue light. This protein acts by regulating the proteasome-dependent degradation of TOC1 protein and its functioning of this protein is required for normal circadian cycle [159]. Its normal functioning is sustained by GI which directly interacts with it via protein–protein interaction. Moreover, the interaction between these two proteins is enhanced in blue light via flavin-binding LIGHT, OXYGEN OR VOLTAGE (LOV) domain of ZTL [160]. Mutations in LOV domain the affects ZTL-GI interaction and results in greatly diminished activity of *ZTL* [160]. Studies have shown that overexpression of *ZTL* significantly delays flowering under long day conditions, and loss-of-function mutation of this gene have a little effect on flowering time [107].

The comparison within structures of flowering genes from five citrus species (sweet orange, clementine, mandarin, citron, and pummelo) showed that the proteins sequences as well as intron-exon organization showed variation (Table 4; Fig. 7). The variation could have been due the dissimilar lengths of introns the sequences. The comparison of genetic and protein structures could be helpful in detecting potential target sequences and residues through genetic engineering tools for generation of mutations at specific locations. The expression analysis of flowering genes revealed that the highest level of expression was observed in bud and ovules (Fig. 10). The qRT-PCR analysis was performed to identify chief flowering genes in *Citrus* (Figs. 11 and 12). The results revealed that *CsFT* was highly expressed in bud tissue as compared to control tissue leaf as well as other flowering and fruit developmental stages. Similar results were reported by Nishikawa et al. [160] who reported negative correlation of *CiFT* mRNA levels with fruit weight per leaf area in case of satsuma mandarin (*Citrus unshiu* Marc.). The results could help in identifying specific tissue for targeting the specific flowering related genes to induce or early flowering. Pajon et al. [161] studied the expression analysis of *CiFT1*, *CiFT2* and *CiFT3* in ‘Pineapple’ sweet orange and pummelo leaf tissues over a period of one year. They observed that the expression level of three genes was at peak during the month of April and subsided after that regardless of the conditions in which they were growing (protected or open field conditions). In *C. unshiu*, three *FT* transcripts, *CiFT1*, *CiFT2*, and *CiFT3* have been identified and characterized [75, 162] of which CiFT1 and CiFT2 are isoforms encoded by the same gene [163] and CiFT3 is considered a better floral-inductive treatment compared to CiFT1 and CiFT2 [164, 165]. Soares et al. [108] developed transgenic “Carrizo” citrange hybrid using *CcFT1* and *CcFT3* (homologs of *FT* in *C. clementine*). The transgenic lines overexpressing *CcFT1* were unable to exhibit flowering, while lines overexpressing *CcFT3* exhibited flowering. Thus, *FT3* could act as potential target for its overexpression in citrus to induce early flowering [108, 165]. The qRT-PCR study revealed that the expression of *CsAP* was higher in all samples as compared to leaf tissue used as control (Fig. 11d). Munoz-Fambuena et al. [166] reported higher expression of *CsAP1* in buds as compared to leaves in ‘Moncada’ mandarin. The *CsSOC* showed a slight decrease in gene expression (~ 0.5 fold change) in bud as compared to leaf (Fig. 11c). Citrus homologue of *SOC1*, *CsSL1* has been reported to show constant and similar gene expression level in leaf and bud tissues [166]. The genes *CsAP* and *CsLFY* were highly expressed in early fruit development stage (FruitS1) as compared to other tissues (Fig. 11d and f). These genes have been reported to determine flower meristem identity and their expression under constitutive promoter is sufficient to promote initiation and development of flowering from shoot apical and axillary meristems [79]. The results revealed that identification of tissue for targeting flowering expression is equally important as identifying the genes related to flowering.

Apart from the primary genes, many subsidiary genes are also involved in regulation of flowering either directly or indirectly. The genetic manipulation of these genes i.e. either overexpress or silence their expression could help in achieving early flowering phenotype in citrus (Table 1). *CsCEN* is known to maintain vegetative axillary meristem indeterminacy in citrus [68]. It antagonizes *Thorn Identity 1* (*TI1*) as it is not expressed thorn meristem. Silencing of its activity causes termination in the activity of stem cells which results in dormant axillary meristems converting into thorns. CsCEN functions in association with CsFLD to repress the expression of *TI1* and mutations in *TI1* and *TI2* could rescue the *cscen* mutant phenotype [68]. Various studies have shown that the loss-of-function mutation of *CEN/TFL1* can result in precocious flowering in fruit crops such as kiwifruit [167], pear [168], apple [168, 169], and blueberry [170]. Another gene *VIN3* is required for the vernalization response in *Arabidopsis*. Plants mutated to silence the activity of this gene are unable to respond to vernalization resulting in increase of *FLC* transcript levels ultimately leading to a late flowering phenotype [71]. DELLA protein is a negative regulator of gibberellic acid signalling which is crucial for flowering under short day conditions [171]. A study in *Arabidopsis* has shown that a quadruple mutant of DELLA develops early flowers under short day conditions [172]. These flowering genes could be targeted for their overexpression or silencing in order to generate desired flowering phenotype in citrus.

## Conclusion

Citrus is an important horticultural crop grown for its high nutritional value. However, the long juvenility period makes it difficult for crop improvement. To break the juvenility period using biotechnological techniques, it is important to understand the genetic makeup of the flowering genes. The present research was carried out to elucidate the structural and functional analysis of flowering genes in sweet orange. A total of 43 flowering genes were identified in sweet orange which were distributed along the 9 chromosomes. The in-silico analysis of the gene and protein sequences revealed the involvement of flowering genes in circadian rhythm pathways regulated by light-receptors cryptochromes and phytochromes. The comparative analysis was carried out among other species of citrus viz., sweet orange, clementine, mandarin, citron and pummelo. Some of the genes shared dissimilar genetic structure but similar protein structure confirming the conserved nature of coding sequences in flowering genes. The expression study revealed that expression of the flowering genes were high in fruit ovule as compared to fruit bud. The qRT-PCR analysis identified the tissue specific expression of flowering genes (*CsFT, CsCO, CsSOC, CsAP, CsSEP* and *CsLFY*) which would help in manipulation of the pathways for in depth understanding of the pathways. The various flowering genes in citrus could be targeted via biotechnological approaches including overexpression, loss-of-mutation, RNA interference and CRISPR-*Cas* technologies. The study could prove useful for genetic manipulation of flowering genes in citrus species.

### Supplementary Information

**Additional file 1: Table S1. **List of primers used in RT-PCR analysis. **Table S2. **Location of CREs on various flowering genes in sweet orange. **Table S3. **GO annotation of flowering genes in sweet orange. **Table S4. **Gene function analysis of flowering genes in sweet orange. **Table S5. **String clustering of the flowering related proteins in sweet orange. **Table S6. **K-mean clustering of the flowering related proteins in sweet orange. **Table S7. **rkpdm values of flowering genes expressed in different tissues of different citrus species. **Fig. S1. **Melt curve of the genes (a) *CsFT* (b) *CsCO* (c) *CsSOC* (d) *CsAP* (e) *CsSEP* (f) *CsLFY.*

## Data Availability

The datasets supporting the conclusion of this article are available in the ‘Citrus Genome Database’ (https://www.citrusgenomedb.org/) under the link (https://www.citrusgenomedb.org/organism/Citrus/sinensis; Gene Sequence IDs provided in Table 1) and ‘Plant Ensembl’ (https://plants.ensembl.org/index.html).

## Abbreviations

CREs: Cis-Regulatory Elements
KEGG: Kyoto Encyclopedia of Genes and Genomes
PEBP: Phosphatidylethanolamine-binding proteins
RNAi: RNA interference
CRISPR/Cas: Clustered regularly interspaced short palindromic repeats
CDS: Coding sequence
GO: Gene ontology
PPI: Protein-protein interaction
