# Supplementary material for: Genome-wide identification and characterization of flowering genes in Citrus sinensis (L.) Osbeck: a comparison among C. Medica L., C. Reticulata Blanco, C. Grandis (L.) Osbeck and C. Clementina
Source: BMC Genom Data. 2024 Feb 20;25:20. doi: 10.1186/s12863-024-01201-5 (PMC10880302; doi:10.1186/s12863-024-01201-5)
Supplement: Supplementary file 1 — Additional file 1: Table S1. List of primers used in RT-PCR analysis. Table S2. Location of CREs on various flowering genes in sweet orange. Table S3. GO annotation of flowering genes in sweet orange. Table S4. Gene function analysis of flowering genes in sweet orange. Table S5. String clustering of the flowering related proteins in sweet orange. Table S6. K-mean clustering of the flowering related proteins in sweet orange. Table S7. rkpdm values of flowering genes expressed in different tissues of different citrus species. Fig. S1. Melt curve of the genes (a) CsFT (b) CsCO (c) CsSOC (d) CsAP (e) CsSEP (f) CsLFY. [file 12863_2024_1201_MOESM1_ESM.zip › Additional file 1.docx]

**Genome-wide identification and characterization of flowering genes in *Citrus sinensis* (L.) Osbeck: A comparison among *C. medica* L., *C. reticulata* Blanco, *C. grandis* (L.) Osbeck and *C. clementina***

Harleen Kaur^1^, Pooja Manchanda^1^*, Gurupkar S. Sidhu and Parveen Chhuneja^1^

*^1^School of Agricultural Biotechnology, College of Agriculture, Punjab Agricultural University, Ludhiana-141001, Punjab, India*

*Corresponding author: [poojamanchanda5@pau.edu](mailto:poojamanchanda5@pau.edu)

Table S1: List of primers used in RT-PCR analysis

| **Gene** | **Primer** | **Sequence (5’-3’)** |
| --- | --- | --- |
| *CsFT* | Forward primer | GAGGAAGCTGAAGAGGAAGTG |
|  | Reverse primer | TTGCATTGTTTGCAGTGCTC |
| *CsCO* | Forward primer | TTATCTGACCATCAGTTTATGCGG |
|  | Reverse primer | TGATAAGCAACAATTCCATCGG |
| *CsSOC* | Forward primer | ATGTTGTGAGGATTGTTGAGG |
|  | Reverse primer | GAGTAGGCATCACCATTTCTG |
| *CsAP* | Forward primer | AACAGTATCAGAACCAACAGCC |
|  | Reverse primer | CCACAATCCCAGATATGCGA |
| *CsSEP* | Forward primer | CTTCTTGGAGAGGATTTGGG |
|  | Reverse primer | GCATATACTGTGTCTTGGTGG |
| *CsLFY* | Forward primer | CCAAGAAGGGTTATCGGAGG |
|  | Reverse primer | ATTCATCGTCATCATTCTCGTC |
| *CsACTIN* | Forward primer | GGCATCACACTTTCTACAATGAG |
|  | Reverse primer | ACACAATACCTGTTGTACGACC |

Table S2: Location of CREs on various flowering genes in sweet orange

Supplementary Table S2.xlx

Table S3: GO annotation of flowering genes in sweet orange

| **SeqName** | **Description** | **#GO** | **GO IDs** | **GO Names** | **Enzyme Codes** | **Enzyme Names** |
| --- | --- | --- | --- | --- | --- | --- |
| *CsFT* | selenoprotein H | 1 | C:GO:0005794 | C:Golgi apparatus |  |  |
| *CsCO* | methylenetetrahydrofolate reductase 2 | 6 | P:GO:0009086; P:GO:0035999; F:GO:0071949; F:GO:0106312; F:GO:0106313; C:GO:0005829 | P:methionine biosynthetic process; P:tetrahydrofolate interconversion; F:FAD binding; F:methylenetetrahydrofolate reductase NADH activity; F:methylenetetrahydrofolate reductase NADPH activity; C:cytosol | EC:1.5.1.20 | Methylenetetrahydrofolate reductase (NAD(P)H) |
| *CsSOC1* | putative transcription elongation factor SPT5 homolog 1 isoform X1 | 8 | P:GO:0006357; P:GO:0006368; P:GO:0006412; P:GO:0032784; F:GO:0003729; F:GO:0003735; C:GO:0005840; C:GO:0032044 | P:regulation of transcription by RNA polymerase II; P:transcription elongation from RNA polymerase II promoter; P:translation; P:regulation of DNA-templated transcription, elongation; F:mRNA binding; F:structural constituent of ribosome; C:ribosome; C:DSIF complex |  |  |
| *CsFLD* | protein FLOWERING LOCUS D | 3 | P:GO:0006325; P:GO:0016570; F:GO:0016491 | P:chromatin organization; P:histone modification; F:oxidoreductase activity | EC:1 | Oxidoreductases |
| *CsLFY* | LEAFY-like protein | 3 | P:GO:0006355; F:GO:0003677; C:GO:0005634 | P:regulation of transcription, DNA-templated; F:DNA binding; C:nucleus |  |  |
| *CsSVP* | MADS-box protein SVP | 5 | P:GO:0045944; F:GO:0000977; F:GO:0003700; F:GO:0046983; C:GO:0005634 | P:positive regulation of transcription by RNA polymerase II; F:RNA polymerase II transcription regulatory region sequence-specific DNA binding; F:DNA-binding transcription factor activity; F:protein dimerization activity; C:nucleus |  |  |
| *CsTFL* | protein TERMINAL FLOWER 1 | 11 | P:GO:0006355; P:GO:0006623; P:GO:0009744; P:GO:0009910; P:GO:0010022; P:GO:0010228; F:GO:0003712; C:GO:0005634; C:GO:0005773; C:GO:0005886; C:GO:0031982 | P:regulation of transcription, DNA-templated; P:protein targeting to vacuole; P:response to sucrose; P:negative regulation of flower development; P:meristem determinacy; P:vegetative to reproductive phase transition of meristem; F:transcription coregulator activity; C:nucleus; C:vacuole; C:plasma membrane; C:vesicle |  |  |
| *CsBFT* | CEN-like protein 1 | 3 | P:GO:0009908; P:GO:0010228; C:GO:0005737 | P:flower development; P:vegetative to reproductive phase transition of meristem; C:cytoplasm |  |  |
| *CsMADS_AGL61* | zinc finger CCCH domain-containing protein 13 | 2 | F:GO:0003677; F:GO:0046872 | F:DNA binding; F:metal ion binding |  |  |
| *CsMADS_AGL82* | pentatricopeptide repeat-containing protein At2g20710, mitochondrial-like | 1 | C:GO:0005739 | C:mitochondrion |  |  |
| *CsMADS_AGL31* | G2/mitotic-specific cyclin-2-like | 7 | P:GO:0000079; P:GO:0044772; P:GO:0051301; F:GO:0016538; C:GO:0000307; C:GO:0005634; C:GO:0005737 | P:regulation of cyclin-dependent protein serine/threonine kinase activity; P:mitotic cell cycle phase transition; P:cell division; F:cyclin-dependent protein serine/threonine kinase regulator activity; C:cyclin-dependent protein kinase holoenzyme complex; C:nucleus; C:cytoplasm |  |  |
| *CsMADS_AGL3* | Developmental protein SEPALLATA 1 | 5 | P:GO:0045944; F:GO:0000978; F:GO:0000981; F:GO:0046983; C:GO:0005634 | P:positive regulation of transcription by RNA polymerase II; F:RNA polymerase II cis-regulatory region sequence-specific DNA binding; F:DNA-binding transcription factor activity, RNA polymerase II-specific; F:protein dimerization activity; C:nucleus |  |  |
| *CsMADS_AGL70* | integrin-linked protein kinase 1 isoform X1 | 4 | P:GO:0006468; F:GO:0004674; F:GO:0005524; C:GO:0016021 | P:protein phosphorylation; F:protein serine/threonine kinase activity; F:ATP binding; C:integral component of membrane | EC:2.7.1 | Transferring phosphorus-containing groups |
| *CsMADS_AGL72* | MADS-box protein AGL42 | 9 | P:GO:0009838; P:GO:0009909; P:GO:0010150; P:GO:0045944; P:GO:0080187; F:GO:0000978; F:GO:0000981; F:GO:0046983; C:GO:0005634 | P:abscission; P:regulation of flower development; P:leaf senescence; P:positive regulation of transcription by RNA polymerase II; P:floral organ senescence; F:RNA polymerase II cis-regulatory region sequence-specific DNA binding; F:DNA-binding transcription factor activity, RNA polymerase II-specific; F:protein dimerization activity; C:nucleus |  |  |
| *CsMADS_AGL35* | agamous-like MADS-box protein AGL80 | 5 | P:GO:0045944; F:GO:0000978; F:GO:0000981; F:GO:0046983; C:GO:0005634 | P:positive regulation of transcription by RNA polymerase II; F:RNA polymerase II cis-regulatory region sequence-specific DNA binding; F:DNA-binding transcription factor activity, RNA polymerase II-specific; F:protein dimerization activity; C:nucleus |  |  |
| *CsTSF* | 50S ribosomal protein 5 alpha, chloroplastic | 2 | P:GO:0032544; C:GO:0009536 | P:plastid translation; C:plastid |  |  |
| *CsAP3* | agamous-like MADS-box protein TM6 | 6 | P:GO:0009908; P:GO:0045944; F:GO:0000978; F:GO:0000981; F:GO:0046983; C:GO:0005634 | P:flower development; P:positive regulation of transcription by RNA polymerase II; F:RNA polymerase II cis-regulatory region sequence-specific DNA binding; F:DNA-binding transcription factor activity, RNA polymerase II-specific; F:protein dimerization activity; C:nucleus |  |  |
| *CsAP2* | ethylene-responsive transcription factor RAP2-7 | 4 | P:GO:0006355; F:GO:0003677; F:GO:0003700; C:GO:0005634 | P:regulation of transcription, DNA-templated; F:DNA binding; F:DNA-binding transcription factor activity; C:nucleus |  |  |
| *CsWUS* | WUSCHEL-related homeobox 3 | 5 | P:GO:0006355; P:GO:0099402; F:GO:0003677; F:GO:0003700; C:GO:0005634 | P:regulation of transcription, DNA-templated; P:plant organ development; F:DNA binding; F:DNA-binding transcription factor activity; C:nucleus |  |  |
| *CsCRY1* | cryptochrome-1 isoform X1 | 10 | P:GO:0006139; P:GO:0006950; P:GO:0009785; P:GO:0032922; P:GO:0043153; F:GO:0003677; F:GO:0009882; F:GO:0071949; C:GO:0005634; C:GO:0005737 | P:nucleobase-containing compound metabolic process; P:response to stress; P:blue light signaling pathway; P:circadian regulation of gene expression; P:entrainment of circadian clock by photoperiod; F:DNA binding; F:blue light photoreceptor activity; F:FAD binding; C:nucleus; C:cytoplasm |  |  |
| *CsCRY2* | cryptochrome-1 isoform X2 | 10 | P:GO:0006139; P:GO:0006950; P:GO:0009785; P:GO:0032922; P:GO:0043153; F:GO:0003677; F:GO:0009882; F:GO:0071949; C:GO:0005634; C:GO:0005737 | P:nucleobase-containing compound metabolic process; P:response to stress; P:blue light signaling pathway; P:circadian regulation of gene expression; P:entrainment of circadian clock by photoperiod; F:DNA binding; F:blue light photoreceptor activity; F:FAD binding; C:nucleus; C:cytoplasm |  |  |
| *CsDL4* | Dicer-like protein 4 |  |  |  |  |  |
| *CsGI* | protein GIGANTEA | 8 | P:GO:0006355; P:GO:0009409; P:GO:0009637; P:GO:0010218; P:GO:0010378; P:GO:0042542; P:GO:0048578; C:GO:0005654 | P:regulation of transcription, DNA-templated; P:response to cold; P:response to blue light; P:response to far red light; P:temperature compensation of the circadian clock; P:response to hydrogen peroxide; P:positive regulation of long-day photoperiodism, flowering; C:nucleoplasm |  |  |
| *CsPHYB* | Phytochrome B | 8 | P:GO:0000160; P:GO:0006355; P:GO:0009584; P:GO:0009585; P:GO:0017006; F:GO:0000155; F:GO:0009881; F:GO:0042803 | P:phosphorelay signal transduction system; P:regulation of transcription, DNA-templated; P:detection of visible light; P:red, far-red light phototransduction; P:protein-tetrapyrrole linkage; F:phosphorelay sensor kinase activity; F:photoreceptor activity; F:protein homodimerization activity | EC:2.7.3; EC:2.7.13.3 | Transferring phosphorus-containing groups; Histidine kinase |
| *CsFLK* | Flowering locus K domain | 5 | P:GO:0009911; P:GO:0010468; F:GO:0003729; C:GO:0005634; C:GO:0005737 | P:positive regulation of flower development; P:regulation of gene expression; F:mRNA binding; C:nucleus; C:cytoplasm |  |  |
| *CsSEP2* | Developmental protein SEPALLATA 1 | 5 | P:GO:0045944; F:GO:0000978; F:GO:0000981; F:GO:0046983; C:GO:0005634 | P:positive regulation of transcription by RNA polymerase II; F:RNA polymerase II cis-regulatory region sequence-specific DNA binding; F:DNA-binding transcription factor activity, RNA polymerase II-specific; F:protein dimerization activity; C:nucleus |  |  |
| *CsPI* | Floral homeotic protein APETALA 3 | 5 | P:GO:0045944; F:GO:0000978; F:GO:0000981; F:GO:0046983; C:GO:0005634 | P:positive regulation of transcription by RNA polymerase II; F:RNA polymerase II cis-regulatory region sequence-specific DNA binding; F:DNA-binding transcription factor activity, RNA polymerase II-specific; F:protein dimerization activity; C:nucleus |  |  |
| *CsMAF1* | protein COFACTOR ASSEMBLY OF COMPLEX C SUBUNIT B CCB1, chloroplastic | 1 | C:GO:0016021 | C:integral component of membrane |  |  |
| *CsSHP1* | Agamous-like MADS-box protein AGL1 | 6 | P:GO:0045944; F:GO:0000978; F:GO:0000981; F:GO:0046983; C:GO:0005634; C:GO:0016021 | P:positive regulation of transcription by RNA polymerase II; F:RNA polymerase II cis-regulatory region sequence-specific DNA binding; F:DNA-binding transcription factor activity, RNA polymerase II-specific; F:protein dimerization activity; C:nucleus; C:integral component of membrane |  |  |
| *CsCEN* | CENTRORADIALIS-like | 4 | P:GO:0009910; P:GO:0010228; C:GO:0005634; C:GO:0005737 | P:negative regulation of flower development; P:vegetative to reproductive phase transition of meristem; C:nucleus; C:cytoplasm |  |  |
| *CsFRI* | FRIGIDA | 2 | P:GO:0009908; P:GO:0030154 | P:flower development; P:cell differentiation |  |  |
| *CsEMF1* | Hypothetical protein CUMW_110510, partial | 2 | P:GO:0009910; P:GO:0045892 | P:negative regulation of flower development; P:negative regulation of DNA-templated transcription |  |  |
| *CsTEM1* | AP2/ERF and B3 domain-containing transcription repressor RAV2-like | 4 | P:GO:0006355; F:GO:0003677; F:GO:0003700; C:GO:0005634 | P:regulation of DNA-templated transcription; F:DNA binding; F:DNA-binding transcription factor activity; C:nucleus |  |  |
| *CsSPB* | Squamosa promoter-binding protein 1-like | 3 | F:GO:0003677; F:GO:0046872; C:GO:0005634 | F:DNA binding; F:metal ion binding; C:nucleus |  |  |
| *CsSPL1* | Squamosa promoter-binding-like protein 1 isoform X1 | 4 | F:GO:0003677; F:GO:0046872; C:GO:0005634; C:GO:0016020 | F:DNA binding; F:metal ion binding; C:nucleus; C:membrane |  |  |
| *CsSPL2* | Squamosa promoter-binding-like protein 2 isoform X1 | 3 | F:GO:0003677; F:GO:0046872; C:GO:0005634 | F:DNA binding; F:metal ion binding; C:nucleus |  |  |
| *CsSUF4* | SUPPRESSOR OF FRI 4 | 2 | F:GO:0003677; F:GO:0046872 | F:DNA binding; F:metal ion binding |  |  |
| *CsVIN3* | OBERON 4 | 5 | P:GO:0010071; P:GO:0010078; P:GO:0010468; P:GO:0010492; C:GO:0005634 | P:root meristem specification; P:maintenance of root meristem identity; P:regulation of gene expression; P:maintenance of shoot apical meristem identity; C:nucleus |  |  |
| *CsVIP3* | WD repeat-containing protein VIP3 | 1 | C:GO:0005634 | C:nucleus |  |  |
| *CsDELLA* | DELLA protein GAIP-B | 13 | P:GO:0006355; P:GO:0009723; P:GO:0009737; P:GO:0009863; P:GO:0009867; P:GO:0009938; P:GO:0010187; P:GO:0042538; P:GO:2000033; P:GO:2000377; F:GO:0003700; F:GO:0043565; C:GO:0005634 | P:regulation of DNA-templated transcription; P:response to ethylene; P:response to abscisic acid; P:salicylic acid mediated signaling pathway; P:jasmonic acid mediated signaling pathway; P:negative regulation of gibberellic acid mediated signaling pathway; P:negative regulation of seed germination; P:hyperosmotic salinity response; P:regulation of seed dormancy process; P:regulation of reactive oxygen species metabolic process; F:DNA-binding transcription factor activity; F:sequence-specific DNA binding; C:nucleus |  |  |
| *CsZTL* | Adagio protein 1 | 10 | P:GO:0009785; P:GO:0009908; P:GO:0010114; P:GO:0010498; P:GO:0016567; P:GO:0043153; F:GO:0009882; C:GO:0005634; C:GO:0005829; C:GO:0019005 | P:blue light signaling pathway; P:flower development; P:response to red light; P:proteasomal protein catabolic process; P:protein ubiquitination; P:entrainment of circadian clock by photoperiod; F:blue light photoreceptor activity; C:nucleus; C:cytosol; C:SCF ubiquitin ligase complex |  |  |
| *CsFT3* | FT-interacting protein 3 | 3 | F:GO:0016757; C:GO:0005783; C:GO:0016020 | F:glycosyltransferase activity; C:endoplasmic reticulum; C:membrane | EC:2.4 | Glycosyltransferases |
| *CsFLC* | MADS-box protein FLOWERING LOCUS C-like isoform X1 | 5 | P:GO:0045944; F:GO:0000977; F:GO:0003700; F:GO:0046983; C:GO:0005634 | P:positive regulation of transcription by RNA polymerase II; F:RNA polymerase II transcription regulatory region sequence-specific DNA binding; F:DNA-binding transcription factor activity; F:protein dimerization activity; C:nucleus |  |  |

Table S4: Gene function analysis of flowering genes in sweet orange

Supplementary Table S4.xlx

Table S5: String clustering of the flowering related proteins in sweet orange

| **#term ID** | **Term Description** | **Observed Gene Count** | **Background Gene Count** | **Strength** | **Matching Proteins In Your Network** |
| --- | --- | --- | --- | --- | --- |
| CL:39848 | Mixed, incl. Transcription factor, MADS-box, and Circadian rhythm - plant | 16 | 106 | 1.99 | CsZTL,CsFLD,CsFLK,CsMADS_AGL35,CsCEN,  CsTFL,CsMADS_AGL72,CsCRY2,CsLFY,CsSVP,  CsDELLA,CsAP3,CsPI,CsPHYB,CsCRY1,CsGI |
| CL:39975 | MADS MEF2-like, and MADS | 10 | 41 | 2.2 | CsFLD,CsFLK,CsMADS_AGL35,CsCEN,CsTFL,  CsMADS_AGL72,CsLFY,CsSVP,CsAP3,CsPI |
| CL:40021 | Mixed, incl. MADS MEF2-like, and Phosphatidylethanolamine-binding, conserved site | 7 | 18 | 2.41 | CsFLD,CsFLK,CsCEN,CsTFL,CsMADS_AGL72,  CsLFY,CsSVP |
| CL:39854 | Circadian rhythm - plant, and DELLA, N-terminal domain superfamily | 6 | 32 | 2.09 | CsZTL,CsCRY2,CsDELLA,CsPHYB,CsCRY1,  CsGI |
| CL:40025 | Mixed, incl. Phosphatidylethanolamine-binding, conserved site, and MADS MEF2-like | 4 | 10 | 2.42 | CsCEN,CsTFL,CsLFY,CsSVP |
| CL:39859 | Circadian rhythm - plant | 4 | 13 | 2.3 | CsZTL,CsCRY2,CsCRY1,CsGI |
| CL:40028 | Mixed, incl. Phosphatidylethanolamine-binding protein, and Plant bZIP transcription factors | 3 | 5 | 2.59 | CsTFL,CsLFY,CsSVP |
| CL:40047 | Mixed, incl. Domain with 2 conserved Trp (W) residues, and SWIRM domain | 3 | 5 | 2.59 | CsFLD,CsFLK,CsMADS_AGL72 |
| CL:39977 | MADS MEF2-like, and MADS | 3 | 19 | 2.01 | CsMADS_AGL35,CsAP3,CsPI |
| CL:39875 | Kelch motif, and Zinc finger protein CONSTANS-like | 2 | 5 | 2.42 | CsZTL,CsGI |
| CL:39898 | DELLA, N-terminal domain superfamily, and Transcription factor PIF3-like | 2 | 10 | 2.12 | CsDELLA,CsPHYB |

Table S6: K-mean clustering of the flowering related proteins in sweet orange

| **Cluster Number** | **Cluster Color** | **Protein Name** | **Protein Identifier** | **Protein Description** |
| --- | --- | --- | --- | --- |
| 1 | Red | CsFRI | 2711.A0A067F2L7 | FRIGIDA-like protein; Belongs to the Frigida family. |
|  |  | CsFT | 2711.A0A067FMA4 | Uncharacterized protein. |
|  |  | CsFT3 | 2711.A0A067FP05 | Uncharacterized protein. |
|  |  | CsMADS_AGL72 | 2711.A0A067F225 | MADS-box domain-containing protein. |
|  |  | CsMADS_AGL82 | 2711.A0A067G5U9 | Uncharacterized protein. |
|  |  | CsSHP1 | 2711.A0A067F470 | MADS-box domain-containing protein. |
|  |  | CsSPL2 | 2711.A0A067GT75 | SBP-type domain-containing protein. |
|  |  | CsSUF4 | 2711.A0A067GC54 | BED-type domain-containing protein. |
|  |  | CsVIN3 | 2711.A0A067GV08 | Uncharacterized protein. |
|  |  | CsVIP3 | 2711.A0A067DQW9 | WD_REPEATS_REGION domain-containing protein. |
| 2 | Yellow | CsBFT | 2711.A0A067EY77 | Uncharacterized protein. |
|  |  | CsCRY1 | 2711.A0A067GZR5 | Photolyase/cryptochrome alpha/beta domain-containing protein. |
|  |  | CsCRY2 | 2711.A0A067F285 | Photolyase/cryptochrome alpha/beta domain-containing protein. |
|  |  | CsGI | 2711.A0A067H897 | Uncharacterized protein. |
|  |  | CsPHYB | 2711.A0A067G8U7 | Phytochrome; Regulatory photoreceptor which exists in two forms that are reversibly interconvertible by light: the Pr form that absorbs maximally in the red region of the spectrum and the Pfr form that absorbs maximally in the far-red region. |
|  |  | CsZTL | 2711.A0A067DQL4 | F-box domain-containing protein. |
| 3 | Green | CsAP3 | 2711.A0A067FUM1 | Uncharacterized protein. |
|  |  | CsCEN | 2711.A0A067ESH5 | Uncharacterized protein. |
|  |  | CsEMF1 | 2711.A0A067GJ69 | Uncharacterized protein. |
|  |  | CsFLC | 2711.A0A067H2B6 | Uncharacterized protein. |
|  |  | CsFLK | 2711.A0A067E4I5 | Uncharacterized protein. |
|  |  | CsLFY | 2711.A0A067F352 | Floricaula/leafy-like transcription factor; Probable transcription factor. Belongs to the FLO/LFY family. |
|  |  | CsPI | 2711.A0A067G526 | Uncharacterized protein. |
|  |  | CsSPB | 2711.A0A067DGL7 | SBP-type domain-containing protein. |
|  |  | CsSVP | 2711.A0A067FAR8 | MADS-box domain-containing protein. |
|  |  | CsTFL | 2711.A0A067F1A2 | Uncharacterized protein. |
| 4 | Cyan | CsCO | 2711.A0A067DZ70 | Methylenetetrahydrofolate reductase; Belongs to the methylenetetrahydrofolate reductase family. |
|  |  | CsFLD | 2711.A0A067E350 | SWIRM domain-containing protein. |
|  |  | CsMADS_AGL3 | 2711.A0A067FEC4 | K-box domain-containing protein. |
|  |  | CsMADS_AGL31 | 2711.A0A067GZE0 | Cyclin N-terminal domain-containing protein; Belongs to the cyclin family. |
|  |  | CsMADS_AGL35 | 2711.A0A067EPZ3 | MADS-box domain-containing protein. |
|  |  | CsMADS_AGL61 | 2711.A0A067F165 | C3H1-type domain-containing protein. |
|  |  | CsMADS_AGL70 | 2711.A0A067GR87 | Uncharacterized protein. |
|  |  | CsSEP2 | 2711.A0A067GAA0 | K-box domain-containing protein. |
|  |  | CsSOC1 | 2711.A0A067GV45 | Transcription elongation factor SPT5. |
|  |  | CsTSF | 2711.A0A067EQE4 | Uncharacterized protein. |
| 5 | Blue | CsAP2 | 2711.A0A067G0B6 | Uncharacterized protein. |
|  |  | CsDELLA | 2711.A0A067FB95 | GRAS domain-containing protein; Belongs to the GRAS family. |
|  |  | CsDL4 | 2711.A0A067F7B2 | Uncharacterized protein; Belongs to the helicase family. Dicer subfamily. |
|  |  | CsMAF1 | 2711.A0A067DSL2 | Uncharacterized protein. |
|  |  | CsTEM1 | 2711.A0A067FRT4 | Uncharacterized protein. |
|  |  | CsWUS | 2711.A0A067G072 | Homeobox domain-containing protein. |
|  |  | CsSPL1 | 2711.A0A067G9I0 | SBP-type domain-containing protein. |

Table S7: rkpdm values of flowering genes expressed in different tissues of different citrus species

| **Gene** | ***C. medica* bud** | ***C. clementina* fruit** | ***C. clementina* ovule** | ***C. unshiu* fruit peel** | ***C. reticulata* ovule** |
| --- | --- | --- | --- | --- | --- |
| *FT* | 49.3368 | 73.0912 | 21.6328 | 5.29998 | 54.2233 |
| *CO* | 63.5438 | 325.518 | 11.1171 | 76.2316 | 22.5065 |
| *SOC1* | 53.2583 | 30.9153 | 126.51 | 36.9892 | 80.2159 |
| *FLD* | 12.2964 | 10.4531 | 30.7867 | 15.1084 | 23.5158 |
| *LFY* | 7.58356 | 0.0577134 | 1.92128 | 0.268542 | 1.32426 |
| *SVP* | 128.926 | 0 | 0 | 0.019793 | 0.155386 |
| *TFL1* | 0 | 0 | 0 | 0 | 0 |
| *BFT* | 1.05641 | 1.12648 | 2.19742 | 0.527548 | 1.20772 |
| *MADS_AGL42* | 0.94944 | 42.3785 | 18.5663 | 2.07611 | 34.0479 |
| *MADS_AGL61* | 44.7257 | 20.5603 | 29.6042 | 15.0178 | 32.1047 |
| *MADS_AGL82* | 0 | 0 | 0 | 0 | 0 |
| *MADS_AGL31* | 2.52628 | 9.77933 | 0 | 0.0824857 | 1.0823 |
| *MADS_AGL3* | 3.78264 | 45.8979 | 2.44443 | 5.69248 | 4.77785 |
| *MADS_AGL13* | 0.94944 | 42.3785 | 18.5663 | 2.07611 | 34.0479 |
| *MADS_AGL70* | 12.3506 | 27.1331 | 108.372 | 22.6938 | 61.6505 |
| *MADS_AGL72* | 145.561 | 0 | 0 | 0.578064 | 0 |
| *MADS_AGL24* | 128.926 | 0 | 0 | 0.019793 | 0.155386 |
| *MADS_AGL35* | 0 | 0 | 0.617156 | 0 | 0 |
| *TSF* | 0.62735 | 14.6758 | 10.6601 | 10.1868 | 27.8387 |
| *AP3* | 5.95632 | 1.19515 | 0.727377 | 28.1186 | 0.902122 |
| *AP2* | 38.2149 | 81.3254 | 68.5125 | 55.1771 | 53.7046 |
| *WUS* | 0 | 0.304748 | 0 | 0 | 0 |
| *CRY1* | 133.683 | 26.7248 | 70.2208 | 17.8561 | 32.4537 |
| *CRY2* | 43.4003 | 55.4755 | 25.9492 | 60.8106 | 25.6763 |
| *DL4* | 13.6856 | 15.1582 | 2.22019 | 12.8649 | 7.30441 |
| *GI* | 6.08074 | 58.1859 | 416.87 | 232.364 | 273.225 |
| *PHYB* | 32.2669 | 17.8 | 3.53956 | 12.4988 | 5.97382 |
| *FLK* | 33.3959 | 49.2755 | 93.8616 | 31.819 | 79.9759 |
| *SEP1* | 0.2359 | 118.84 | 206.466 | 237.41 | 330.449 |
| *PI* | 5.95632 | 37.1343 | 0.957777 | 7.82291 | 7.86545 |
| *MAF1* | 18.306 | 9.81935 | 7.22473 | 17.2355 | 6.2724 |
| *SHP1* | 0 | 30.7599 | 37.7854 | 68.2739 | 55.927 |
| *DELLA* | 57.1418 | 26.3220 | 130.8423 | 31.0734 | 71.1400 |
| *SUF4* | 62.4098 | 33.2067 | 96.2060 | 54.0150 | 57.4718 |
| *SPB* | 45.7041 | 18.5933 | 1.5567 | 18.0300 | 9.8900 |
| *ZTL* | 22.9345 | 19.9297 | 35.7867 | 68.9050 | 30.2867 |
| *EMF1* | 6.1425 | 3.9233 | 13.9787 | 2.7450 | 5.0133 |
| *VIP3* | 58.4807 | 49.0367 | 17.3600 | 45.6450 | 44.1867 |
| *VIN3* | 52.5256 | 20.6833 | 38.0233 | 23.8600 | 22.3800 |
| *SPL2* | 29.6311 | 32.7900 | 22.5117 | 18.9150 | 8.9733 |
| *FLC* | 220.8040 | 10.1800 | 12.0433 | 12.0400 | 22.9000 |
| *TEM1* | 106.4540 | 107.8833 | 299.8900 | 187.1200 | 199.7633 |
| *FRI* | 12.1076 | 9.0333 | 19.0767 | 9.6650 | 12.7833 |
| *FT3* | 6.0254 | 49.5733 | 0.3667 | 31.4200 | 4.0933 |
| *CEN* | 0.0000 | 5.1467 | 0.4133 | 0.6150 | 0.0000 |
| *SPL1* | 63.6868 | 38.8133 | 147.7877 | 59.7100 | 82.1900 |


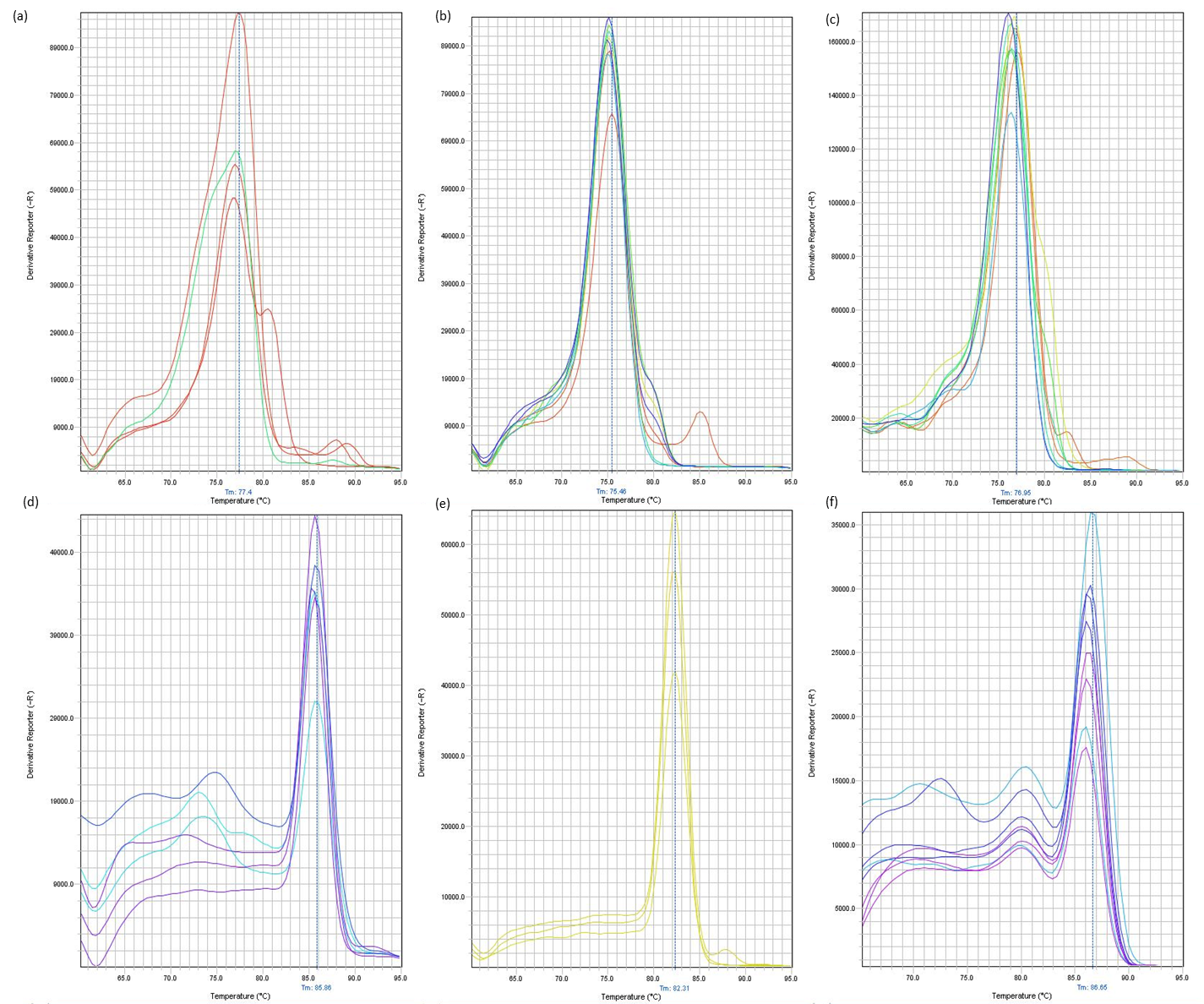


Fig.S1 Melt curve of the genes (a) *CsFT* (b) *CsCO* (c) *CsSOC* (d) *CsAP* (e) *CsSEP* (f) *CsLFY.*
